# Does concern about falling predict future falls in older adults? A systematic review and meta-analysis

**DOI:** 10.1093/ageing/afaf089

**Published:** 2025-04-08

**Authors:** Toby Jack Ellmers, Jodi P Ventre, Ellen Freiberger, Klaus Hauer, David B Hogan, Mei Ling Lim, Lisa McGarrigle, Samuel Robert Nyman, Chris J Todd, Yuxiao Li, Kim Delbaere

**Affiliations:** Department of Brain Sciences, Faculty of Medicine, Imperial College London, London, UK; National Institute for Health and Care Research, Applied Research Collaboration- Greater Manchester & School of Health Sciences, Faculty of Biology, Medicine and Health, The University of Manchester, Manchester, UK; Institute for Biomedicine of Aging, FAU Erlangen-Nürnberg, Nuremburg, Germany; Bethanien Hospital, Geriatric Centre at the Heidelberg University, Heidelberg 69126, Germany; Professor Emeritus, Departments of Medicine and Community Health Sciences, Cumming School of Medicine, University of Calgary, Calgary, Canada; Falls, Balance and Injury Research Centre, Neuroscience Research Australia, Sydney, Australia & School of Population Health, University of New South Wales, Sydney, Australia & Neurology, The George Institute for Global Health, Sydney, Australia; School of Health Sciences, Faculty of Biology, Medicine and Health, The University of Manchester, Manchester, UK; Department of Psychology, Faculty of Humanities & Social Sciences, University of Winchester, Winchester, UK; National Institute for Health and Care Research, Applied Research Collaboration - Greater Manchester & School of Health Sciences, Faculty of Biology, Medicine and Health, The University of Manchester, Manchester, UK/& Manchester Academic Health Science Centre, Manchester, UK & Manchester University NHS Foundation Trust; Department of Brain Sciences, Faculty of Medicine, Imperial College London, London, UK; Falls, Balance and Injury Research Centre, Neuroscience Research Australia, Randwick, NSW 2031, Australia & School of Population Health, University of New South Wales, Sydney, Australia

**Keywords:** fear of falling, falls efficacy, balance confidence, fall risk assessment, systematic review, older people

## Abstract

**Background:**

The 2022 World Falls Guidelines recommend assessing concerns (or ‘fears’) about falling in multifactorial fall risk assessments. However, the evidence base for this recommendation is limited. This review evaluated the evidence for concerns about falling as an independent predictor of future falls, applying the Bradford Hill criteria for causality.

**Methods:**

Systematic review and meta-analyses were conducted (PROSPERO registration ID: CRD42023387212). MEDLINE, CINAHL Plus, Web of Science and PsycINFO were searched for studies examining associations between baseline concerns about falling and future falls in older adults (minimum 6-month follow-up). Meta-analyses examined associations between concerns about falling and future falls. Risk of bias was assessed using an adapted Newcastle Ottawa Scale for cohort studies, and evidence certainty was rated with GRADE.

**Results:**

53 studies, comprising 75,076 participants, were included. Meta-analysis showed significant independent association between baseline concerns and future falls when using the Falls Efficacy Scale-International to assess concerns (full scale version, pooled OR = 1.03 [95% CI = 1.02–1.05] per 1-point increase; short scale version, pooled OR = 1.08 [95% CI = 1.05–1.11]). Significant associations were also observed when using single-item measures of concerns (pooled OR = 1.60 [95% CI = 1.36–1.89] for high vs. low concerns). In contrast, balance confidence (Activities-Specific Balance Confidence Scale) did not predict future falls (pooled OR = 0.97 [95% CI = 0.93–1.01]). Despite 26 studies rated as poor quality, associations were consistent across studies of different quality. The overall certainty of the evidence was rated as moderate.

**Conclusions:**

Baseline concern about falling is a clear predictor of future falls in older adults, supporting its inclusion in fall risk assessments. Regular assessment of concerns about falling, along with targeted interventions, could help reduce the risk of falls in older adults.

## Key Points

- This systematic review and meta-analysis examined the association between baseline concerns about falling and future falls.
- Concerns about falling were a clear independent predictor of future falls.
- Consistent results were observed across study designs, research settings and measurement tools.
- Balance confidence did not predict future falls.
- Clinicians should regularly screen for concerns about falling, as targeting them could reduce falls.

## Introduction

Falls are a major global public health challenge and a leading cause of disability and mortality in older adults [1], costing the US healthcare system an estimated $50 billion annually [2]. With an ageing population, falls demand urgent attention from healthcare providers and policymakers. Many older adults develop concerns (or ‘fears’) about falling [3, 4], which affect up to 50% of those aged 60 years and above depending on the population and assessment used [4].

The 2022 World Falls Guidelines recommend assessing concerns about falling in multifactorial fall risk assessments of older adults [5]. This was based on evidence linking these concerns to reduced quality-of-life and independence [6], poorer rehabilitation outcomes [7], and increased risk of future physical and social frailty, disability and institutionalisation [7, 8]. Concerns about falling may also increase the likelihood of future falls [9, 10]. However, existing reviews on this topic have primarily focused on cross-sectional studies rather than systematically examining if baseline concerns about falling predict future falls, which precludes causal inference. Further, recent conceptual frameworks [11–13] and epidemiological data [14] also suggest that some level of concern could have protective effects with respect to fall risk (e.g. reduced risk-taking behaviours). There is therefore the need to support or refine the recommendations presented in 2022 World Falls Guidelines surrounding concerns about falling and future falls.

The aim of this systematic review and meta-analysis is to evaluate whether baseline concerns about falling independently predict future falls in older adults. Using the Bradford Hill criteria [15] in our search strategy, we focus on the *Temporality* criterion by including only studies where concerns about falling were measured before the index incident falls. In our interpretation of results, we will focus on the *Strength of Association* and its *Consistency* across studies. Furthermore, we will consider *Experimental Evidence* in our discussion to strengthen the causal inference*.*

## Materials and methods

### Registration and protocol

The study was prospectively registered in PROSPERO (registration ID: CRD42023387212) and followed the Updated Preferred Reporting Items for Systematic Reviews and Meta-Analyses (PRISMA 2020) guidelines [16]. The primary research question was, ‘Does baseline concern about falling predict future falls and/or injurious falls, independent of other known fall risk factors?’

### Search strategy and information sources

We systematically searched MEDLINE, CINAHL Plus, Web of Science and PsycINFO from inception to 6 March 2024 using MeSH terms and keywords related to concerns (and fear) about falling, falls, older people, risk or prospective studies. The detailed search strategy is provided in Supplementary Appendix A.

### Eligibility criteria and study selection

We included articles that explored the association between baseline concerns about falling (or related constructs, e.g. balance confidence) and future falls over a follow-up period of at least 6-months (to ensure sufficient follow-up duration). We included studies that recruited either community-dwelling and/or institutionalised older adults, aged 60 years or older. Validated questionnaires (e.g. variants of the Falls Efficacy Scale–International (FES-I) [17, 18]) and/or single-item measurements (e.g. ‘Are you afraid of falling?’ [19]) were accepted for assessing concern, fear or worries about falling. As there is little difference in fall rates recorded in research using ongoing or recall assessment [20], future falls during the follow-up were either assessed on an ongoing basis (e.g. falls diaries/calendars, regular telephone calls etc.) or at the end of the follow-up period (via recall) [20]. No specific restrictions were applied based on the definition of a fall; studies could report associations between concerns about falling and either the occurrence of falls or the rate of falls. We excluded cross-sectional studies, intervention studies, case reports, reviews, qualitative studies, conference abstracts and letters to the editor, and those not published in the English language. Studies using measures of fear-related activity avoidance as a predictor variable, or those focusing exclusively on participants with specific diseases or conditions (e.g. dementia, Parkinson’s Disease, stroke etc.), were also excluded. Titles, abstracts and full texts were reviewed by two independent reviewers (a combination of either T.J.E., J.V. or E.F.) with disagreements resolved following discussions with a third neutral reviewer. Covidence software was used for article management.

### Data extraction

Data were extracted independently by two reviewers (T.J.E. and either J.V. or K.D.), with a third neutral reviewer resolving any discrepancies through discussion and consensus. Extracted data included study characteristics and sample details, methods and effect estimates. Critical information that remained missing following emails to the authors led to study exclusion (e.g. measurement of concerns), while non-critical missing data were noted as ‘not reported’.

Concern about falling and balance confidence were treated as distinct constructs [11], and studies were classified accordingly based on the assessment tools used. Fall outcomes were categorised into any-type falls (occurrence of at least one fall, injurious or not, during follow-up), recurrent falls (≥2 falls) or injurious falls (see Supplementary Appendix B description of specific falls outcomes used in each study) [21].

### Risk of bias and quality assessment

Following the registration of the protocol, we decided to change the risk of bias tool from ROBINS-E to an adapted version of the Newcastle-Ottawa Scale. This decision was made following pilot assessments conducted within the team, in which the ROBINS-E was deemed unsuitable for the types of studies included in this review (see also [22]). Risk of bias was assessed by two independent reviewers (a combination of J.V., M.L.L., S.N. and E.F.) using an adapted version of the Newcastle Ottawa Scale for cohort studies [23]. (See Supplementary Appendix G for the specific version of the scale used.) This scale assesses risk of bias due to cohort selection, confounding, measurement of exposure (concern about falling tool) and outcome (falls, including follow-up period), and incomplete or missing outcome data. Inter-rater agreement was 94.3%, and disagreements were resolved by consensus with a third neutral reviewer (T.J.E.). Studies were rated as ‘good’, ‘fair’ or ‘poor’ quality (see Supplementary Appendix G).

### Grading of recommendations, assessment, development and evaluation

The certainty of the evidence was assessed by two independent reviewers (L.M and T.J.E.) using the GRADE approach (high, moderate, low, very low) [24] for prognostic factors and future outcomes [25, 26]. The following factors were considered in relation to downgrading the evidence: (i) risk of bias, (ii) inconsistency, (iii) imprecision (i.e. do 95% CIs overlap unity?), (iv) indirectness and (v) publication bias. No upgrades were applied. As this was a prognostic review, the starting point for assessment was an initial assumption of high certainty evidence [25, 26]. Initial inter-rater agreement was 85%; any disagreements were resolved by consensus with a third neutral reviewer (J.V.).

### Statistical analysis

Meta-analyses were pre-planned to evaluate the effect of concerns about falling on future falls, with separate meta-analyses conducted for different assessment tools (concerns about falling: FES-I, Short FES-I and single-item assessments; balance confidence: Activities-Specific Balance Confidence Scale (ABC)). Due to insufficient data and substantial heterogeneity in defining recurrent or injurious falls, statistical analyses focused on studies reporting any-type falls only.

Two-tailed random effects models were used (α = 0.05). To ensure consistency in the meta-analyses, we focused on odds ratio (OR) as this was the most commonly reported effect size, with 95% confidence intervals (CI) as the measure of association. Only studies reporting ORs and 95% CIs (or providing them upon request) were included in the meta-analyses. For FES-I and ABC, ORs were extracted when these variables were analysed continuously (with ORs reflecting one-point increase on these scales), while for single-item assessments, ORs were extracted for dichotomised groups (e.g. high vs. low concerns). As we wished to explore if concerns about falling independently predicted future falls, adjusted ORs were pooled when available; with adjustments for age, sex, previous falls at baseline and/or physical function (see Supplementary Appendix C for the full list of adjusted variables for each study). If a study presented multiple adjusted ORs (or both adjusted and unadjusted ORs), we selected the maximally adjusted model for pooling. Unadjusted ORs were included for studies without adjustment for confounding. Pre-planned subgroup and sensitivity analyses were conducted to explore the impact of confounding variables, excluding extreme outliers [27] and varying risk of bias [28]. Heterogeneity was assessed using the Cochrane’s Q test and *I*^2^ statistic, with tau^2^ as an estimator of between-study variance. Publication bias was evaluated through Egger’s asymmetry test and visual inspection of a funnel plot for meta-analyses with at least 10 studies [29]. All analyses were performed using R (version 4.3.1) and the ‘metafor’ package (version 4.2.0).

A narrative review was conducted for outcomes with substantial heterogeneity, such as recurrent and injurious falls, synthesising patterns and differences qualitatively without meta-analysis.

## Results

### Study selection and characteristics

**Figure 1:**
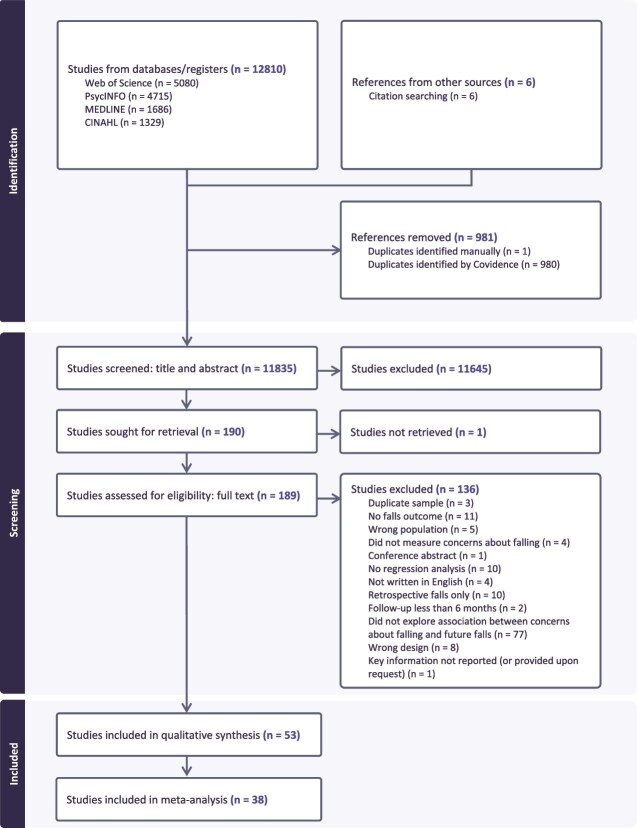
PRISMA flow diagram of the identification, screening and eligibility of included articles.

The PRISMA flow diagram (Figure 1) shows the selection process. After removing duplicates, 11,835 articles were screened and 189 reviewed as full-text. Fifty-three articles met the inclusion criteria [9, 10, 14, 30–79], with 38 included in the meta-analyses [9, 10, 14, 32, 33, 35–45, 51–56, 58, 59, 62, 63, 65–68, 70–72, 74, 76, 77, 79]. If multiple articles used the same dataset, those with a higher risk of bias were excluded (e.g. Chen and Kim [80] excluded, with Okoye et al. [55] included).

Supplementary Appendix B shows the study characteristics of the included studies. The included studies involved 75,076 participants, with sample sizes ranging from 42 [59] to 22,533 [14], and a median of 461. The percentage of females ranged between 30% [54] and 100% [34, 61, 79], with a median of 60.3%. The mean age of participants in individual studies ranged between 65 and 80 years, with three studies reporting a mean age of 83 years [40, 59, 77]. The proportion of fallers (any-type falls) ranged from 9.0% [48] to 72.3% [75], with a median of 33.4% (see Supplementary Appendix D). Most participants were community-dwelling, with only four studies recruiting from residential care [59, 63, 67, 70]. Most of the studies took place in Europe (*N* = 20) or North America (*N* = 14), followed by Asia (*N* = 8), Australia (*N* = 7), and South America (*N* = 3), with one article studying participants in both Asia and Australia [43]. All studies adopted a prospective design to explore if baseline concerns predicted future falls. One study adopted a prospective case-controlled design [49], whilst all others were prospective cohort studies. Follow-up periods ranged from 6 months [38, 42, 45, 52, 61, 62, 67, 70, 77] to 11 years [78], with a median of 1 year.

### Assessment of concerns about falling

The most common method to assess concerns about falling was a single-item question (*N* = 24), with the most common question being ‘Are you afraid of falling?’ (*N* = 14). These dichotomised participants into, e.g. ‘high’ vs. ‘low’ concerns (see Supplementary Appendix B). The FES-I was used in 23 studies, which included either the full (*N* = 16), short (*N* = 6), or iconographic versions (Icon-FES; *N* = 1). Balance confidence was assessed in 16 studies using either ABC (*N* = 9) or FES (*N* = 7).

### Assessment of future falls

Thirty-eight studies assessed any-type falls, and 13 assessed recurrent falls (*N* = 13), comparing these to either non-fallers (*N* = 6 [10, 31, 37, 41, 60, 63]) or a combined group of non-fallers and single fallers (*N* = 7 [32, 43, 44, 47, 48, 57, 64]). Six papers studied injurious falls [49, 65, 69, 71, 73, 78] and 2 studied ‘serious falls’ (≥1 injurious fall and/or ≥2 non-injurious falls [32, 47]). One paper assessed the occurrence of a single fall [60].

### Risk of bias and quality assessment

Twenty-five studies were rated good quality [9, 10, 14, 30, 34, 38, 40, 42, 44, 48, 50, 53–57, 63, 65, 68–71, 73–75], two as fair [58, 66], and 26 as poor [31–33, 35–37, 39, 41–43, 47, 49, 51, 52, 59–62, 64, 67, 72, 76–79, 81]. Poor quality was attributed to single-item questionnaires (*N* = 19), lack of controlling for important confounding variables (*N* = 20), fall assessments recorded through recall at the end of follow-up (*N* = 18), and inadequate follow-up of participants (*N* = 16). Only 17 studies were awarded the maximum 2 stars for the ‘confounding variables’ domain [14, 30, 36, 40–42, 44, 50, 55, 56, 63, 68–70, 72, 73, 81]. (See Supplementary Appendix H for risk of bias and quality assessment for individual studies.)

### Associations with future any-type falls (combined single, recurrent and injurious falls)

#### Concerns about falling: multi-item assessments (FES-I)

**Figure 2:**
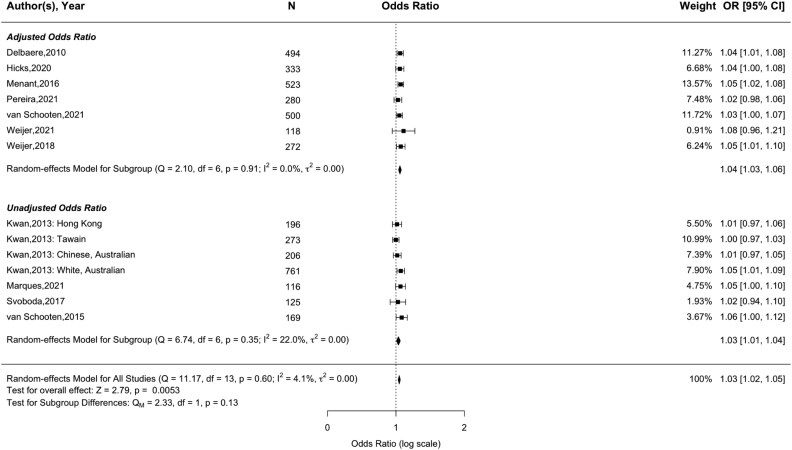
Forest plot of the association between the full 16-item Falls Efficacy Scale International (FES-I) and future any-type falls.

**Figure 3:**
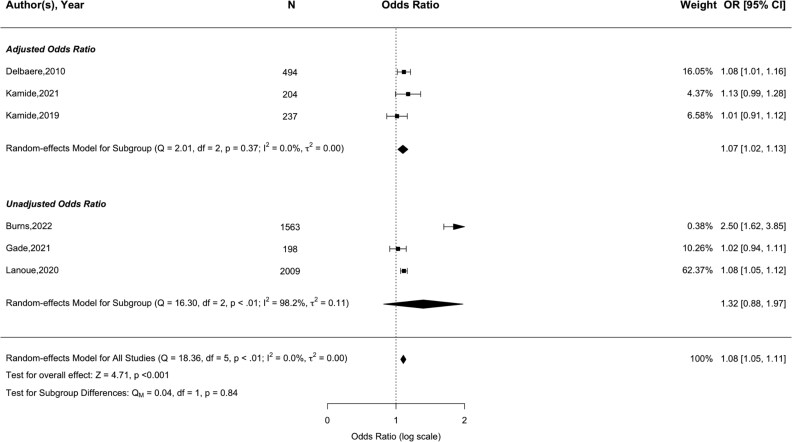
Forest plot of the association between short 7-item Falls Efficacy Scale International (FES-I) and future any-type falls.

Sixteen papers assessed the association between concerns about falling and future falls, using either the FES-I (*N* = 10) [43, 52, 53, 56, 62, 67, 68, 70, 71, 82], Short FES-I (*N* = 5) [35, 41, 42, 45, 76] or both (*N* = 1) [32]. Meta-analyses showed significant associations for both the FES-I (pooled OR = 1.03, 95% CI = 1.02–1.05, *Z* = 2.79, *P* = .005) and Short FES-I (pooled OR = 1.08, 95% CI = 1.05–1.11, *Z* = 4.71, *P* < .001). These results remained when removing an extreme outlier within the Short FES-I analysis (pooled OR = 1.07, 95% CI = 1.05–1.10, *Z* = 4.44, *P* < .001; see Supplementary Appendix F1). No evidence of publication bias (Supplementary Appendix E1; Z = 0.99, *P* = .32) or heterogeneity was found (FES-I, *I*^2^ = 4.1%, *P* = .60; Short FES-I (after adjusting for outlier), *I*^2^ = 0.1%, *P* = .44). Study quality varied, with no significant differences observed when analysing based on either risk of bias (FES-I, *P* = .28; Short FES-I, *P* = .45 (see Supplementary Appendix F3 and F4)) or adjustment for confounding variables (FES-I, *P* = .13 (see Figure 2); Short FES-I, *P* = .84 (see Figure 3)).

#### Concerns about falling: single-item assessments

**Figure 4:**
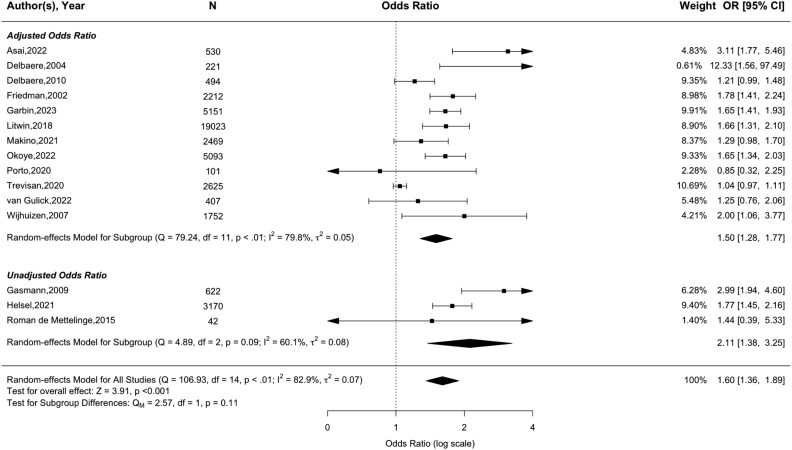
Forest plot of the association between single-item measures of concerns about falling and future any-type falls.

Eighteen studies used single-item assessments to evaluate concerns about falling and future any-type falls [9, 10, 14, 30, 32, 36, 37, 39, 50, 51, 55, 58, 59, 63, 66, 72, 74, 81]. A meta-analysis based on 15 of these studies (adjusted, *N* = 12 [9, 10, 14, 32, 36, 51, 55, 58, 63, 66, 72, 74]; unadjusted, *N* = 3 [37, 39, 59]), comprising 43,912 participants (Figure 4), showed that concerns about falling were significantly associated with future falls (pooled OR = 1.60, 95% CI = 1.36–1.89, *Z* = 3.91, *P* < .01). Despite substantial heterogeneity (*I*^2^ = 82.9%, *P* < .01), results remained robust after outlier removal (pooled OR = 1.58, 95% CI = 1.34–1.86, *Z* = 3.96, *P* < .001; see Supplementary Appendix F2). There were no significant subgroup differences based on either risk of bias (*P* = .22 (see Supplementary Appendix F5)) or adjustment for confounding variables (*P* = .11 (see Figure 4)). According to the funnel plot (see Supplementary Appendix E2) and Egger test, there was no evidence for significant publication bias (Z = 1.75, *P* = .08).

#### Balance confidence

**Figure 5:**
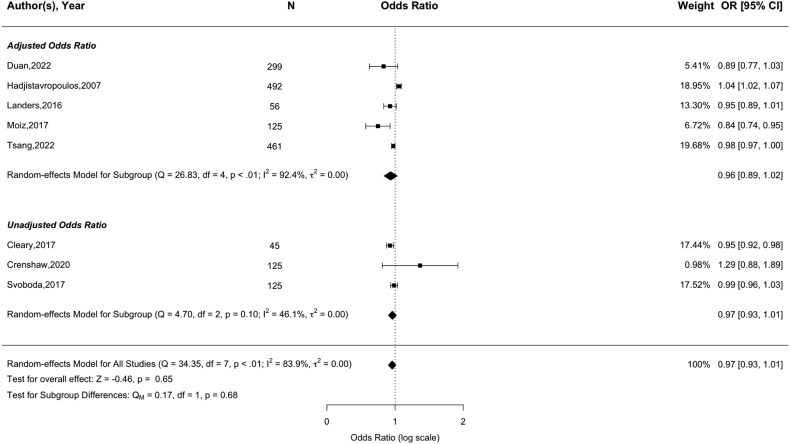
Forest plot of the association between balance confidence (Activities-Specific Balance Confidence Scale [ABC]) and future any-type falls.

Thirteen papers assessed the association between balance confidence and future falls, using either the ABC scale (*N* = 9) [33, 38, 44, 50, 54, 62, 65, 77, 79] or FES (*N* = 6) [30, 38, 44, 61, 64, 75]. Meta-analysis of nine studies using the ABC (five adjusted [33, 38, 44, 54, 65] and three unadjusted studies [62, 77, 79]), comprising 1728 participants, found no significant association with future falls (pooled OR = 0.97, 95% CI = 0.93–1.01, *Z* = −0.46, *P* = .65; Figure 5). There was evidence of substantial heterogeneity (*I*^2^ = 83.9%, *P* = .01). No significant differences emerged when analysing by risk of bias (*P* = .89 (see Supplementary Appendix F6)) or adjustment for confounding variables (*P* = .68 (see Figure 5)). Six papers assessed balance confidence using the original FES. It was not possible to conduct a meta-analysis for this data due to substantial heterogeneity with scoring systems used. Four papers reported a significant association (adjusted, *N* = 3 [30, 38, 75]; unadjusted, *N* = 1 [64]), whilst two reported a lack of association (adjusted, *N* = 1 [44]; unadjusted, *N* = 1 [61]).

#### GRADE certainty of the evidence

The GRADE summary of findings is presented in Appendix H. For all analyses, the overall certainty of the evidence was moderate. Meta-analysis for FES-I, Short FES-I and single item assessments was downgraded due to the risk of bias (primarily due to a lack of controlling for confounding variables and issues with fall assessment and data completeness). Results for balance confidence (ABC) were downgraded due to imprecision.

#### Associations with future recurrent and injurious falls

Due to substantial heterogeneity with respect to assessing both predictor and outcome variables, recurrent and injurious falls are analysed via narrative review only. Nine studies assessed the association between concerns about falling and recurrent falls with mixed results. Two studies found a significant association between the FES-I and future recurrent falls in adjusted models [32, 83]. Another study found a significant association in unadjusted models for White Australians but not for those from Asian backgrounds [43]. In adjusted models, one study found that Short FES-I scores predicted future recurrent falls [32], whereas another study reported a non-significant association [41]. When using single-item measures, two adjusted [48, 63] and two unadjusted studies [37, 64] found significant associations, but one adjusted study did not [32]. Regarding balance confidence, the ABC showed a significant association with recurrent falls [44], as did a modified version of the FES [57]. However, the original FES did not [44].

Similar mixed results were observed for the four studies investigating the association with future injurious falls. Using a single-item measure, one study found a significant association in an unadjusted analysis [78], while another [73] observed a lack of significant association in an adjusted analysis. Using a case-controlled design, a study found that a single-item measure independently predicted more serious injuries following a fall (fractures vs. soft-tissue damage) [49]. In contrast, another reported a non-significant association when using the FES-I in an adjusted model [71]. Two studies investigated the association between balance confidence (ABC, *N* = 1 [65]; FES, *N* = 1 [69]) and found no significant association with future injurious falls in adjusted models.

## Discussion

The 2022 World Falls Guidelines recommended assessing concerns about falling as part of a multifactorial falls risk assessment [5], based on intermediate evidence (GRADE 1B). By applying the Bradford Hill criteria [15], we provide stronger causal evidence, upgrading the evidence to strong (GRADE 1A—using the same modified GRADE approach as used in the World Falls Guidelines [5]). These findings emphasise the clinical importance of assessing concerns about falling.

Significant associations between concerns about falling and future falls were consistently found across all measures (short/full FES-I and single-item measures), supporting a causal relationship (Bradford Hill criteria [15]). Concerns about falling may reflect a sensitive self-perception of fall risk, complementing objective markers like prior falls or gait speed. Pooled ORs were 1.03 (95% CI = 1.02–1.05) for the FES-I and 1.08 (95% CI = 1.05–1.11) for the Short FES-I, per point increase. Whilst the strength of these associations depends on scale used and the points accrued, scoring 40 points higher on the FES-I or 15 points higher on the Short FES-I corresponds to a 120% increase in the odds of falling, emphasising the *Strength of the Association* [15]. Although formal comparisons of diagnostic accuracy between measures are beyond the scope of this study, it is worth noting that this would be double the strength of the association observed with dichotomised single-item measures (pooled OR = 1.60 [95% CI = 1.36–1.89]), which also showed considerable heterogeneity (*I*^2^ = 82.9%). In contrast, the FES-I or short FES-I showed low heterogeneity in predicting future falls, and can also provide additional insights into an individual’s concerns about falling not afforded when using single-item measures (i.e. magnitude and specificity rather than mere presence). These findings therefore support the use of the FES-I (full or short) for assessing concerns about falling, in line with the 2022 Guidelines [5, 12]. However, single-item measures remain a practical alternative for time-constrained clinical settings [84].

*Experimental Evidence* (in-line with the Bradford Hill criteria [15]) further supports a causal association, showing that greater concerns predict more falls during lab-based testing [85, 86], and lead to overly-cautious and potentially unsafe walking behaviours [87, 88]. There is also some evidence that psychological interventions targeting concerns about falling can reduce incidence of falls [89]. While previous reviews have identified prior falls as a risk factor for developing concerns about falling [4], our findings reveal a bidirectional relationship: concerns about falling can be both a cause and consequence of falls. Interestingly, our review found that concerns about falling specifically, rather than balance confidence, were associated with future falls. This distinction indicates that balance confidence should not be part of standardised fall risk assessments. While related, balance confidence and concerns about falling are distinct constructs [11]. Our findings emphasise this distinction, revealing that people with low confidence, but no concerns, are not necessarily at higher risk of falling.

We used a comprehensive search strategy, incorporating data from 53 studies with 75,076 participants across multiple regions, enhancing the *Consistency* of our findings [15]. We minimised bias by focusing on prospective studies with follow-up periods of 6 months or longer, stratifying our meta-analysis by assessment tools, risk of bias and confounding variables. Despite these strengths, our review has several limitations. There was substantial heterogeneity existed across studies, particularly in the confounding variables, participant inclusion/exclusion criteria, and fall assessment. Most studies excluded people with significant cognitive or neurological impairments, limiting generalisability. It should, however, be noted that *Consistent* evidence of a relationship between baseline concerns and future falls has been reported across different populations, including people with Stroke [90], Parkinson’s Disease [91] and Multiple Sclerosis [68, 92]. Most included studies also focused exclusively on people living in the community, with only four studies recruiting participants from residential care [59, 63, 67, 70]. While three of these studies reported significant associations between concerns and future falls, further research is required to explore how concerns about falling affect fall risk in older adults residing in long-term care. Finally, the small number of studies on recurrent or injurious falls and the heterogeneity in their assessment precluded meta-analysis for these outcomes. More prospective research is needed to evaluate the relationship between concerns about falling and future recurrent or injurious falls.

There are also methodological limitations of the studies reviewed. Many studies (*N* = 37) did not control for crucial confounding factors, which could impact the *Strength of Association* and the validity of causal inferences (Bradford Hill criteria [15]). To address this limitation, we separated our meta-analyses into adjusted and unadjusted results, observing statistically comparable results when doing so. Other limitations included high attrition rates (14 studies lost >20% of participants) and non-gold standard approaches to assessing falls (17 studies used recall at the end of the follow-up). Despite these issues, no significant differences were observed when grouping studies by overall quality, which considered statistical power, data missingness and fall assessment methods, suggesting a robust underlying association between concerns about falling and future falls.

Our findings fill a gap in evidence, supporting the inclusion of concerns about falling in fall risk assessments in national [93, 94] and international [5] guidelines. We recommend using the short 7-item FES-I in clinical settings, in line with the 2022 World Falls Guidelines [5], due to its brevity (<3 min to complete), excellent psychometric properties [18], and validated cut-off points for identifying high concerns (≥11/28) [95]. Our pooled results indicate that people scoring above this cut-off have between 32% and 168% greater odds of falling (depending on their specific score) compared to an individual scoring the minimum points (7/28), reflecting a substantial *Strength of Association* between concerns about falling and future falls.

## Conclusions

Concerns about falling in older adults are highly prevalent [4] and have been linked to reduced quality of life and independence [6], poorer rehabilitation outcomes [7] and increased risk of frailty, disability and institutionalisation [7, 8]. Our review identifies that concerns about falling can lead to a further negative outcome: increased future falls. We therefore recommend that clinicians regularly assess concerns about falling in all community-dwelling older adults. Identified concerns should be managed with a multidisciplinary approach including exercise, cognitive behavioural therapy and/or occupational therapy, as recommended in the 2022 World Falls Guidelines [5].

## Supplementary Material

aa-24-2050-File003_afaf089

## Research Data Transparency and Availability:

All extracted data from included studies (including all data analysed within the meta-analysis) is available within the Supplementary Materials. Code used to analyse the data is available from the corresponding author upon reasonable request.
