# Correction to: Does concern about falling predict future falls in older adults? A systematic review and meta-analysis

**DOI:** 10.1093/ageing/afaf183

**Published:** 2025-06-18

This is a correction to: Toby Jack Ellmers, Jodi P Ventre, Ellen Freiberger, Klaus Hauer, David B Hogan, Mei Ling Lim, Lisa McGarrigle, Samuel Robert Nyman, Chris J Todd, Yuxiao Li, Kim Delbaere, Does concern about falling predict future falls in older adults? A systematic review and meta-analysis, *Age and Ageing*, Volume 54, Issue 4, April 2025, afaf089, https://doi.org/10.1093/ageing/afaf089

In the originally published version, the third author's affiliation was listed incorrectly and has been updated.

