# Supplementary material for: Does concern about falling predict future falls in older adults? A systematic review and meta-analysis
Source: Age Ageing. 2025 Apr 8;54(4):afaf089. doi: 10.1093/ageing/afaf089 (PMC11976718; doi:10.1093/ageing/afaf089)
Supplement: aa-24-2050-File003_afaf089 [file aa-24-2050-file003_afaf089.docx]

**Concerns about falling as a predictor of future falls in older adults: A systematic review and meta-analysis**

**Appendix A.** Search strategy

**Appendix B.** Study characteristics and key data

**Appendix C.** Adjusted covariates in included studies

**Appendix D.** Proportion and/or rate/number of falls in each study

**Appendix E.** Funnel plot of Meta-analyses

**Appendix F.** Sensitivity and subgroup analyses

**Appendix G.** Risk of bias scoring system

**Appendix H.** Risk of bias assessment

**Appendix I.** GRADE certainty of evidence scoring

**Appendix J.** PRISMA guidelines checklist

**Appendix A. Search strategy**

| **Database** | **Search strategy** |
| --- | --- |
| **MEDLINE (Ovid)** | Ovid MEDLINE(R) and Epub Ahead of Print, In-Process, In-Data-Review & Other Non-Indexed Citations and Daily <March 23, 2023>  1 Older adult/  2 (senior* or elder* or aged* or older person* or older people or gerontological or geriatric or 60 years old).mp.  3 1 or 2  4 Fear of falling/  5 (falls efficacy or balance confidence or fall related efficacy or activity restriction or concern* about falling or {*FES*}).mp.  6 4 or 5  7 Fall/  8 (accidental fall* or injurious fall* or fall injury*).mp.  9 7 or 8  10 Prospective/  11 (prospective cohort or prospective cohort study* or longitudinal or longitudinal study* or cohort or risk or risk factor* or predict*).mp.  12 10 or 11  13 3 and 6 and 9 and 12 1686 |
| **CINAHL** | S1 MH “Older adult”  S2 Senior* or elder* or aged* or older person* or older people or gerontological or geriatric or 60 years old  S3 S1 OR S2  S4 MH “Fear of falling”  S5 Falls efficacy or balance confidence or fall related efficacy or activity restriction or concern* about falling or {*FES*}  S6 S4 OR S5  S7 MH “Fall”  S8 Accidental fall* or injurious fall* or fall injury*  S9 S7 OR S8  S10 MH “Prospective”  S11 Prospective cohort or prospective cohort study* or longitudinal or longitudinal study* or cohort or risk or risk factor* or predict*  S12 S10 OR S11  S13 S3 AND S6 AND S9 AND S12 1329 |
| **PsycINFO**  **(Ovid)** | APA PsycInfo <March 2023>  1 Older adult/  2 (senior* or elder* or aged* or older person* or older people or gerontological or geriatric or 60 years old).mp.  3 1 or 2  4 Fear of falling/  5 (falls efficacy or balance confidence or fall related efficacy or activity restriction or concern* about falling or {*FES*}).mp.  6 4 or 5  7 Fall/  8 (accidental fall* or injurious fall* or fall injury*).mp.  9 7 or 8  10 Prospective/  11 (prospective cohort or prospective cohort study* or longitudinal or longitudinal study* or cohort or risk or risk factor* or predict*).mp.  12 10 or 11  13 3 and 6 and 9 and 12 5715 |
| **Web of Science (Clarivate)** | (Older adult OR senior* OR elder* OR aged* OR older person* OR older people OR gerontological OR geriatric OR 60 years old) AND (Fear of falling OR falls efficacy OR balance confidence OR fall related efficacy OR activity restriction OR concern* about falling OR {*FES*}) AND (Fall OR accidental fall* OR injurious fall* OR fall injury*) AND (Prospective OR prospective cohort OR prospective cohort study* OR longitudinal OR longitudinal study* OR cohort OR risk OR risk factor* OR predict*) 6373 |

| Appendix B. Study characteristics and key data. | | | | | | | | | |
| --- | --- | --- | --- | --- | --- | --- | --- | --- | --- |
| Study (author and year) | **Location & setting** | **Sample size** | ***n* used in main analysis [*n* missing]** | **Age and gender** | **Concern about falling tool** | **Population (inclusion/ exclusion)** | **Falls outcome, definition and assessment method** | **Length of follow-up** | **Main findings from statistical tests (effect size, CIs and p-values)^a^** |
| Allali et al. (2017) | USA, community | 449 | 449 [0] | Mean age = 76.5 yrs ± 6.6  Female = 56.8% | ABC (0-100%; higher scores = greater balance confidence); analysed as a continuous variable  Single-item question: “Do you have a FOF?” Yes/No | **Inclusion:** Aged ≥65 yrs  **Exclusion:** Dementia, significant loss of vision or hearing, inability to ambulate independently, and current or past history of neurological or psychiatric disorders or medical procedures that may affect mobility | **Outcome:** Any-type falls  **Falls definition:** “Unintentionally coming down to the floor or lower level not due to a major intrinsic or extrinsic event.”  **Method:** Recorded via telephone and/or in-person interviews every 2-3 months | Mean follow-up: 20.1 ± 12.2 mnths (range = 1.4 − 43.5 mnths) | ABC  **Unadjusted HR =** 0.98 [95% CI = 0.97–0.99], *p*  < .001  **Adjusted HR** = 0.99 [95% CI = 0.98–1.00], *p* = .040  Single-item assessment  **Unadjusted HR =** 1.75 [95% CI = 1.25–2.43], *p* < .001  **Adjusted HR** = 1.38 [95% CI = 0.94–2.00], *p* = .101 |
| Aoyama et al. (2010) | Japan, geriatric outpatient clinic (but all community-dwelling) | 59 | 58 [1] | Mean age = 80.5 yrs ± 5.7  Female = 100% | FES (10-100; higher scores = lower falls efficacy/ confidence); analysed as a continuous variable | **Inclusion:** Female patients aged ≥65 yrs who were attending the Geriatric Outpatient Clinic of Nagoya University Hospital.  **Exclusion:** hospital admission within 6 months; uncontrolled hypertension; dementia; ischemic heart disease or heart failure; chronic obstructive pulmonary disease; acute orthopedic pain and presence of neurological impairments, and; low fall-risk (scoring 5 or less on the Fall Risk Index) | **Outcome:** Any-type falls  **Falls definition:** “An unintentional change in body position resulting in contact with the ground or with another lower level, however, not as a result of a major intrinsic event (e.g. stroke, syncope) or an overwhelming hazard (e.g. car accident)”  **Method:** Recorded via a falls diary (instructed to record any fall that occurred) | 6 mnths | **Unadjusted OR:** 1.05 [95% CI = 0.97–1.13], *p* = .249 |
| Asai et al. (2022) | Japan, community | 801 | 530 [271] | 65-69 yrs = 12.1%; 70-74 yrs = 35.3%; 75-79 yrs = 30.2%; 80-84 yrs = 16.4%; 85+ yrs = 6.0%  Female = 66.8% | Single-item question: “Are you afraid of falling?”  Yes/No | **Inclusion:** Aged ≥65 yrs and ability to walk independently with/without an assistive device.  **Exclusion:** Cognitive impairment (rapid dementia screening test score<8); self-reported neurological disease (stroke and Parkinson’s disease), and; missing data. | **Outcome:** Any-type falls  **Falls definition:** “An event that resulted in the participant unintentionally coming to the ground or another lower level”  **Method:** Collected at follow-up, retrospectively | 1 yr | **Unadjusted RR** = 3.34 [95% CI = 2.27–5.07]  **Adjusted RR** = 3.70 [95% CI = 2.48–5.67]  **Adjusted OR** = 3.11 [95% CI = 1.80–5.54]^††^  *^††^Data provided by authors, on request* |
| Burns et al. (2022) | USA, community | 1905 | 1563 [342] | 65-74 yrs = 68.2%; 75-84 yrs = 26.5%; 85+ yrs = 5.3%  Female = 52.5% | Short FES-I (7-28; higher scores = greater concerns about falling); analysed as a continuous variable | **Inclusion:** Aged ≥65 yrs; speaks English, and; able to pass a brief verbal memory three-word recall by correctly recalling all three words. | **Outcome:** Any-type falls  **Falls definition:** “An event that resulted in a person unintentionally coming to rest on the ground, floor, or other lower level”.  **Method:** Monthly fall surveys. | 1 yr | **Unadjusted OR** = 2.50 [95% CI = 1.60–3.80] |
| Cleary & Skornyakov (2017) | USA, community | 46 | 45 [1] | Mean age = 83.2 yrs ± 6.3  Female = 68.9% | ABC (0-100%; higher scores = greater balance confidence); analysed as a continuous variable | **Inclusion:** Aged ≥65 yrs; ability to provide informed consent, and; able to ambulate inside their homes (with or without an assistive device).  **Exclusion:** Those who required physical assistance from another person to walk within their homes. | **Outcome:** Any-type falls  **Falls definition:** “Unintentionally coming to the floor, ground or other lower level.”  **Method:** Telephone/in-person interviews every 3 months. | 6 mnths | **Unadjusted OR** = 0.95 [95% CI = 0.92–0.99], *p* = .010. |
| Clemson et al. (2015) | Australia, community | 1000 | 904 [96] | Mean age = 73.4 yrs (range = 65-94)  Female = 53.3% | Single-item question: “Are you afraid of falling?”  No fear (“not at all afraid”) vs. fear (“somewhat afraid”, “fairly afraid”, or “very afraid”). | **Inclusion:** Aged ≥65 yrs, and; living in the community.  **Exclusion:** Living in non-private accommodation; could not speak conversational English, and; could not be interviewed at home for health reasons. | **Outcome:** Injurious falls (a fall requiring medical treatment)  **Falls definition:** Any fall in which they received medical treatment from injuries.  **Method:** Face-to-face interviews, every 2 years (across the 11 yr follow-up) | 11 yrs; but falls assessed every 2 yrs | **Unadjusted HR** = 1.61, *p* = .012^††^  *††As the significance level was above the cut-off required to enter the variable into the full model (p = .010), FOF was not entered into the adjusted model.* |
| Crenshaw et al. (2020) | USA, community | 125 | 125 [0] | Mean age = 77.1 yrs ± 7.5  Female = 100% | ABC (0-100%; higher scores = greater balance confidence); analysed as a continuous variable | **Inclusion:** Aged ≥65 yrs; female; ability to walk a city block without a gait aid; no previous diagnosis of dementia, and; cognitively intact. | **Outcome:** Any-type falls  **Falls definition:** “When the participant lost their balance and landed 1) on the floor, ground, or lower level; 2) on an object (e.g. furniture); or 3) against a wall or railing.”  **Method:** Twice monthly questionnaires. | 1 yr | **Unadjusted OR** = 1.29 [95% CI = 0.89–1.92], *p* = .20 |
| Cumming et al. (2000) | Australia, recruited via hospitals (but all community-dwelling) | 528 | 418 [110] for the FES analysis.  528 [0] for single-item FOF analysis. | Mean age = 77 yrs  Female = 57% | FES (0-100; higher scores = greater falls efficacy/ confidence); split into high (100/100), medium (76-99/100) and low (≤75/100) falls efficacy  Single item question: “Are you afraid of falling?”  Yes/No | **Inclusion:** Aged ≥65 years; living in the community (not a nursing home or hostel for the aged) in the Central Sydney Area Health Service region.  **Exclusion:** Inpatients were excluded if a home visit by an occupational therapist was planned as part of their usual care.  *Note: Persons with cognitive impairment were included as long as they lived with someone who was able to give informed consent and who could report on falls during follow-up.* | **Outcome:** Any-type falls  **Falls definition:** No information provided on how a fall was defined.  **Method:** Collected via monthly fall calendars (completed each day). | 1 yr | FES  **Medium falls efficacy (vs. high falls efficacy):** Unadjusted HR = 1.70 [95% CI = 1.16–2.49]; Adjusted HR = 1.49 [95% CI = 1.01–2.20]  **Low falls efficacy (vs. high falls efficacy):** Unadjusted HR = 2.90 [95% CI = 1.91–4.40]; Adjusted HR = 2.09 [95% CI = 1.31–3.33]  Single-item assessment  **Unadjusted HR =** 1.48 [95% CI = 1.12–1.95]  **Adjusted HR** = 1.21 [95% CI = 0.90–1.62] |
| de Souza et al. (2019) | Brazil, community | 705 | 345 [360] | 60-75 yrs = 71.0%; 75+ yrs = 29.0%  Female = 65.2% | Brazilian FES-I (16-64; higher scores = greater concerns about falling); analysed as a continuous variable | **Inclusion:** Aged ≥60 yrs; resident in the urban area (community); have no cognitive decline, and; ability to walk, allowed to use a walking aid device (cane, crutch or walker).  **Exclusion**: Participant not contactable after three attempts by the interviewer; change of city, and; hospitalized and with neurological diseases that hinder evaluations. | **Outcome:** Single falls (fell once) and recurrent falls (2 or more falls)  **Falls definition:** No information provided on how falls were defined  **Method:** Collected retrospectively at follow-up assessment | 2 yrs | **Outcome:** Single fall (versus no fall)  **Unadjusted OR =** 1.03 [95% CI = 1.00–1.05=, *p* = .065  **Adjusted OR =** 1.01 [95% CI = 0.98–1.04], *p* = .586  **Outcome:** Recurrent fall (versus no fall)  **Unadjusted OR** = 1.07 [95% CI = 1.05–1.10], *p*  < .001  **Adjusted OR =** 1.05 [95% CI = 1.03–1.08], *p*  < .001 |
| Delbaere et al. (2004) | Belgium, community | 225 | 221 [4] | Mean age = 72.0 yrs ± 5.6  Female = 58.4% | Single-item question: “In general, are you afraid of falling?”  ‘No fear’ ("No, not at all") vs ‘Fear’ = ("A little", "quite a bit" or "very much so") | **Inclusion:** Aged ≥60 yrs, and; living in the community.  **Exclusion:** Musculoskeletal problems such as amputation; acute or terminal illness, and; prior severe central nervous system involvement. | **Outcome:** Any-type falls and recurrent falls (those with faller status at baseline and 1+ fall during follow-up)  **Falls definition:** “An unintentional change in body position resulting in contact with the ground or with another lower level, however not as a result of a major intrinsic event (e.g. stroke, syncope) or an overwhelming hazard (e.g. car accident).”  **Method:** Assessed via monthly fall calendars. | 1 yr | **Outcome:** Any-type falls  **Adjusted OR** = 12.33 [95% CI = 1.56–97.54], *p* = .017.  **Outcome:** Recurrent falls (vs. no falls)  **Unadjusted OR** = 2.83 [95% CI = 1.78–4.52], *p* < .001. |
| Delbaere et al. (2006) | Belgium, community | 263 | 257 [6] | Mean age = 72.1 yrs ± 5.5  Female = 56.1% | Single-item question: “In general, are you afraid of falling?”  ‘No fear’ ("No, not at all") vs ‘Fear’ = ("A little", "quite a bit" or "very much so") | **Inclusion:** Aged ≥60 yrs, and; community dwelling.  **Exclusion:** Inability to walk because of musculoskeletal problems; acute or terminal illness, and; documented severe disorders of the central nervous system (e.g. major stroke, Parkinson's disease, Alzheimer's disease). However, a subject with a previous history of stroke could be included provided he or she had recovered with no cognitive impairment or other residual effects that would affect the study assessments. | **Outcome:** Recurrent falls (those with faller status at baseline and 1+ fall during follow-up) vs. no falls  **Falls definition:** “An unintentional change in position resulting in coming to rest on the ground or another lower level, and not as a result of a major intrinsic event (e.g. stroke, syncope) or overwhelming hazard (e.g. car accident).”  **Method:** Assessed via monthly falls calendars. | 1 yr | **Unadjusted OR** = 3.25 [95% CI = 1.86–5.66), p < .001 |
| Delbaere et al. (2010) | Australia, community | 500 | 494 [6] | Mean age = 77.9 yrs ± 4.6  Female = 54.0% | FES-I (16-64; higher scores = greater concerns about falling) and Short FES-I (7-28); analysed as a continuous variable  Single-item question: “Are you afraid of falling?”  No (‘Not at all’) vs. Yes (‘A little bit’, ‘moderately’, ‘quite a lot’, ‘extremely’) | **Inclusion:** Aged 70-90 yrs and community-dwelling.  **Exclusion:** Neurological, cardiovascular, or major musculoskeletal impairments (determined at a baseline assessment) that precluded participants walking 20 m without a walking aid, and; cognitive impairment determined by a score of less than 24 on the Mini-Mental State Examination. | **Outcome:** Any-type falls (1+), recurrent falls (2+), ‘Serious fall’ (experiencing ≥1 injurious fall or ≥2 non-injurious fall)  **Falls definition:** “An unexpected event in which the person comes to rest on the ground, floor, or lower level.”  **Method:** Assessed via monthly diaries. | 1 yr | FES-I  **Outcome:** Serious falls (vs. no falls and single fallers  **Unadjusted OR** = 1.05 [95% CI = 1.02–1.08], *p* = .001  **Adjusted OR =** 1.29 [95% CI = 1.06–1.57], *p* = .010  **Outcome:** Any-type falls vs. no falls  **Unadjusted OR** = 1.05 [95% CI =1.02–1.08], *p* = .002^††^  **Adjusted OR =** 1.04 [95% CI = 1.01–1.08], *p* = .006^††^  **Outcome:** Recurrent falls (vs. no falls and single fallers)  **Unadjusted OR** = 1.06 [95% CI = 1.03–1.10], *p* < .001^††^  **Adjusted OR =** 1.05 [95% CI = 1.02–1.09], *p* = .002^††^  Short FES-I  **Outcome:** Any-type falls vs. no falls  **Unadjusted OR** = 1.10 [95% CI = 1.03–1.17], *p* = .004^††^  **Adjusted OR =** 1.08 [95% CI = 1.01–1.16), *p* = .019^††^  **Outcome:** Recurrent falls (vs. no falls and single fallers)  **Unadjusted OR** = 1.10 [95% CI = 1.03–1.18], *p* = .004^††^  **Adjusted OR =** 1.09 [95% CI = 1.01–1.17], *p* = .024^††^  Single-item assessment  **Outcome:** Any-type falls vs. no falls  **Unadjusted OR** = 1.26 [95% CI = 1.04–1.54], *p* = .020^††^  **Adjusted OR =** 1.21 [95% CI = 0.99–1.48], *p* = .067^††^  **Outcome:** Recurrent falls vs. no falls  **Unadjusted OR** = 1.29 [95% CI = 1.04–1.62], *p* = .024^††^  **Adjusted OR =** 1.22 [95% CI = 0.97–1.54], *p* = .088^††^  *^††^Data provided by authors, on request* |
| Duan et al. (2022) | China, community | 320 | 299 [21] | Mean age = 67.2 yrs ± 6.8  Female = 70.9% | ABC (0-100%; higher scores = greater balance confidence); analysed as a continuous variable | **Inclusion:** Community-dwelling; aged ≥60 yrs; clear consciousness, and; no communication disorders.  **Exclusion:** Severe cardiopulmonary dysfunction; musculoskeletal diseases; neurological dysfunction such as sensory impairment or motor paralysis; and cognitive or psychological impairment. | **Outcome:** Any-type falls  **Falls definition:** “An accident that causes a person to inadvertently lie on the floor or other lower level”  **Method:** No information provided on how self-reported fall data was collected. | 1 yr | **Unadjusted OR** = 0.81 [95% CI = 0.70–0.93], *p* <.001  **Adjusted OR =** 0.89 [95% CI = 0.72–0.96], *p* = .012 |
| Faulkner et al. (2009) | USA, community | 9704 | 8378 [1326] | 65-74 yrs = 75.6%; 75-84 yrs = 22.6%; 85+ yrs = 1.8%  Female = 100% | Single-item question: “Do you have any fear of falling?”  Yes/No | **Inclusion:** Community-dwelling; aged ≥65 yrs; female; Caucasian; able to walk without assistance of another person, and; without hip replacements bilaterally. | **Outcome:** Fall rates (falls divided by follow-up duration)  **Definition:** “Landing on the floor or ground, or falling and hitting an object like a table or a chair”  **Method:** Assessed via postcards and telephone calls every 4 mnths. | 4 yrs | **Adjusted RR (Model 1)** = 1.37 [95% CI = 1.27–1.47]  **Fully adjusted RR (Model 2)** = 1.20 [95% CI = 1.11–1.29] |
| Friedman et al. (2002) | USA, community | 2520 | 2211 [309] | Mean age = 72.6 yrs (range = 65.9–86.3)  Female = 58.6% | Single-item question: “Apart from being in a high place, in the past 12 months, have you been worried or afraid that you might fall?”  Yes/No | **Inclusion:** Aged 65-84 yrs, and; Mini-Mental State Examination score of 18 or higher. | **Outcome:** Any-type falls  **Falls definition:** “Have you fallen within the past 12 months? Falling includes unintentionally coming to rest on the ground or other level such as a chair.”  **Method:** Assessed retrospectively, at follow-up | 20 mnths | **Adjusted OR** = 1.78 [95% CI = 1.41–2.24] |
| Gade et al. (2021) | Denmark, community | 241 | 198 [43]*  **Note, some baseline data missing for 15 participants; random forest imputation used.* | Age, median [IQR] 82 yrs [80 – 86]  Female = 66.4% | Short FES-I (7-28; higher scores = greater concerns about falling); analysed as a continuous variable | **Inclusion:** Community-dwelling, aged ≥75 yrs.  **Exclusion:** Living in care facilities, the presence of self-reported acute illness within seven days before recruitment, being unable to stand for one minute without any assistive device or support from another person, unable to understand Danish, or having a dementia diagnosis. | **Outcome:** Fall rates and occurrence of any-type falls  **Falls definition:** “An unexpected event in which the participants come to rest on the ground floor or lower level”  **Method:** Falls calendars with a daily recording of falls, returned monthly by post. Telephone calls made when calendars were not received, or when a fall occurred to ensure it met the definition of a fall listed above. | 1 yr | **Unadjusted IRR** = 1.06 [95% CI = 1.01–1.11], *p* <.05  **Unadjusted OR** = 1.02 (95% CI = 0.94–1.11), *p* = 0.66^††^  *^††^Data provided by authors, on request* |
| Garbin et al. 2023 | USA, community | 8245 | 5151 [3094] | Mean age = 76.7 yrs ± 7.5  Female = 57.3% | Single-item question: “In the last month, did you worry about falling down?"  Yes/No | *Data obtained from Round 1 (2011) and Round 2 (2012) of the National Health and Aging Trends Study (NHATS)*  **Inclusion:** Aged ≥65 yrs; enrolled to Medicare (which provides healthcare for 96% of Americans aged ≥65 yrs)  **Exclusion:** Not living independently, any missing data. | **Outcome:** Any-type falls  **Falls definition:** “Any fall, slip, or trip in which you lose your balance and land on the floor or ground or at a lower level.”  **Method:** Asked retrospectively, at follow-up | Approx. 1 yr | **Unadjusted OR** = 2.48 [95% CI 2.19–2.82]^††^  **Adjusted OR** = 1.65 [95% CI 1.41–1.93]^††^  *^††^Data provided by authors, on request* |
| Gasmann et al. (2009) | Germany, community | 1801 | 622 [1179] | 65-69 yrs = 32.6%; 70-79 yrs = 50.8%; 80-89 yrs = 14.8% 90 yrs = 1.8%  Female = 48.0% | Single-item question: “Are you afraid of falling?”  Yes/No | Aged ≥65 yrs and community dwelling, living in the metropolitan area of Erlangen, Nuremberg, or Fuerth (Southern Germany) | **Outcome:** Any-type falls: at least 1 fall in the 6-months before the follow-up assessment  Single fall: only 1 fall in 6-months prior to follow-up  Recurrent falls: 2+ falls in 6-months prior to follow-up  **Falls definition:** No information on falls definition provided.  **Method:** Occurrence of falls in the past 6 months was asked retrospectively, during follow-up. | 2 yrs; but falls only assessed in prior 6 mnths | **Outcome:** Any-type falls (vs. no falls):  **Unadjusted OR** = 2.99 [95% CI = 1.95–4.61], *p*<.001  **Outcome:** Single fall (vs. no falls):  **Unadjusted OR =** 2.02 [95% CI = 1.20–3.39], *p*=.007  **Outcome:** Recurrent falls (vs. no falls):  **Unadjusted OR =** 6.67 [95% CI = 3.13–14.18], *p*<.001 |
| Hadjistavropoulos et al. (2007) | Canada, community | 571 | 492 [79] | Mean age = 76.6 yrs ± 5.4  Female = 67.0% | FES (0-10; higher scores = greater falls efficacy/confidence)  ABC (0-100%; higher scores = greater balance confidence)  Both analysed as continuous variables | **Inclusion:** ≥69 years, retired, living in a metropolitan Canadian city. | **Outcome:** Any-type falls  **Falls definition:** No information on falls definition provided  **Method:** A monthly falls diary. Participants had pre-paid postcards and were instructed to report each fall as soon as it occurred. Falls postcards prompted a call to inquire about the nature of the fall. | 6 mnths | FES:  **Adjusted OR =** 0.56 [95% CI = 0.42–0.75]  ABC:  **Adjusted OR =** 1.04 [95% CI = 1.01–1.06] |
| Helsel et al. (2021) | USA, community | 3170 | 3170 [0] | 65-69 yrs = 22.9%; 70-74 yrs = 22.8%; 75-79 yrs = 21.7%; 80-84 yrs = 18.7%; 85+ yrs = 18.7%  Female = 58.4% | Single-item question: “In the last month, did you worry about falling down?" Yes/No | *Data obtained from Round 1 (2011) and Round 4 (2014) of the National Health and Aging Trends Study (NHATS).*  **Inclusion:** Aged ≥65 yrs; community dwelling  **Exclusion:** Data unavailable, lived in a nursing home or unspecified residential facility, or had a proxy respond to the survey with insufficient fall risk information | **Outcome:** Any-type falls  **Falls definition:** “Any fall, slip, or trip in which you lose your balance and land on the floor or ground or at a lower level.”  **Method:** Asked retrospectively | 4 yrs; but falls only assessed in prior 12 mnths | **Unadjusted OR =** 1.77 [95% CI = 1.45–2.16], *p* < .001 |
| Hicks et al. (2020) | Australia, community | 333 | 333 [0] | Mean age = 83.3 yrs ± 4.1  Female = 52.3% | FES-I (16-64; higher scores = greater concerns about falling); analysed as a continuous variable. | **Inclusion:** Aged 70-90 yrs, community dwelling  **Exclusion:** Scored less than 24 in the Mini-Mental State Examination, had insufficient knowledge of English language, presence of a medical or psychological conditions that may have prevented them from completing assessments, or previous diagnosis of dementia or developmental disability, psychotic symptoms, Parkinson’s disease, multiple sclerosis, motor neuron disease or central nervous system inflammation | **Outcome:** Any-type falls  **Falls definition:** “An unexpected event in which the person comes to rest on the ground, floor, or lower level”  **Method:** Monthly fall calendars, with follow-up calls made if fall calendars were not returned | 1 yr | **Unadjusted OR =** 1.06 [95% CI = 1.03–1.09], *p* < .001^††^  **Adjusted OR =** 1.04 [95% CI = 1.00–1.09], *p* = .035^††^  *^††^Data provided by authors, on request* |
| Kamide et al. (2019) | Japan, community | 519 | 237 [282] | Mean age = 71.4 yrs ± 4.6  Female = 75.9% | Short FES-I (7-28; higher scores = greater concerns about falling); analysed as a continuous variable | **Inclusion:** Aged ≥65 yrs, able to perform ADL independently, and able to independently attend the location of the research centre located in the sports facility  **Exclusion:** Suspected dementia, no follow-up data | **Outcome:** Number of falls; occurrence of any-type falls (1+) and recurrent falls.  **Falls definition:** “Unintentionally coming to rest on the ground, the floor, or other lower level”  **Method:** Self-report questionnaire, completed every 6 months at a health check-up | 1 yr | **Outcome:** Number of falls  **Adjusted RR (Model 1) =** 1.09 [95% CI = 1.03–1.15], *p*=.001  **Adjusted RR (Model 2) =** 1.08 [95% CI = 1.01–1.16], *p*=.018  **Outcome:** Any-type falls (vs no falls)  **Adjusted OR (Model 1) =** 1.00 [95% CI = 0.92–1.10], *p* = .920^††^  **Adjusted OR (Model 2) =** 1.01 [95% CI = 0.91–1.12], *p* = .888^††^  **Outcome:** Recurrent falls (vs. no falls)  **Adjusted OR (Model 1) =** 1.13 [95% CI = 1.00–1.27], p = .056^††^  **Adjusted OR (Model 2) =** 1.16 [95% CI = 0.97–1.38] *p* = .106^††^  *^††^Data provided by authors, on request* |
| Kamide et al. (2021) | Japan, community | 265 | 204 [61] | Mean age = 72.9 yrs ± 5.1  Female = 62.3% | Short FES-I (7-28; higher scores = greater concerns about falling); analysed as low (<13 points) vs. high (≥13 points) concerns, and a continuous variable. | **Inclusion:** Age ≥65 yrs, living in the community, and independent in activities of daily living  **Exclusion:** Non-community dwelling, judged as having a care level for certification for long-term care insurance, severe cardio-pulmonary disease or neurological disease, and limitations preventing them from participating in the gait and physical function tests | **Outcome:** Any-type falls  **Falls definition:** “Unintentionally coming to rest on the ground, floor, or other lower level.”  **Method:** Retrospectively recorded at the end of the follow-up | 6 mnths | FES-I, dichotomised:  **Adjusted OR** = 2.72 [95% CI = 1.05–7.06], *p* = .039  FES-I, continuous:  **Adjusted OR** = 1.13 (95% CI: 1.00–1.29, *p* = .058)^††^  *^††^Data provided by authors, on request* |
| Kwan et al. (2013) | ***ChopStix cohorts:*** Taiwan, Hong Kong and Chinese-Australian; community. ***White cohort:*** Australia; Community | 1456 | 1389 [69], fall rates  1436 [20], any-type falls & recurrent falls | Taiwanese cohort, mean age = 74.9 yrs ± 6.4  Hong Kong cohort, mean age = 74.9 yrs ± 6.7  Chinese Australian cohort. mean age = 74.5 yrs ± 6.2  White Australian cohort, mean age = 77.6 yrs ± 4.7  Female = 57.8% | FES-I (16-64; higher scores = greater concerns about falling); analysed as a continuous variable | **Inclusion:** Aged ≥65 yrs (ChopStix cohorts) or ≥70 yrs (White cohort), living independently in the community, able to converse in Chinese (ChopStix cohorts) or English (White cohort)  **Exclusion:** Blindness, being chair bound, suffering from an unstable medical condition, or having a cognitive impairment (Mini-Mental State Examination score of <24 for White cohort, or <19 for ChopStix cohorts, to account for lower literacy) | **Outcome:** Fall rates, and occurrence of any-type falls (1+) and recurrent falls (2+)  **Falls definition:** “Unintentionally coming to the ground or other lower level and other than a consequence of sustaining a violent blow, loss of consciousness, sudden onset of paralysis as in stroke or epileptic seizure”  **Method:** Monthly telephone calls (ChopStix cohorts), and monthly falls diaries and follow-up telephone calls as required (White cohort) | ChopStix cohort = 2 yrs; White cohort = 1 yr | **Outcome:** Fall rates  **Adjusted IRR (Model 1)** = 0.99 [95% CI = 0.98–1.00]  **Adjusted IRR (Model 2) =** 1.03 [95% CI = 1.01–1.05]  **Outcome:** Any-type falls (vs. no falls)  **Unadjusted OR, White Australian** = 1.05 [95% CI = 1.01–1.09]^††^  **Unadjusted OR, Chinese Australian** = 1.01 [95% CI = 0.97–1.05] ^††^  **Unadjusted OR, Hong Kong =** 1.01 [0.97–1.06] ^††^  **Unadjusted OR, Taiwan =** 1.00 [0.97–1.03] ^††^  **Outcome:** Recurrent falls (vs. no falls or single fall)  **Unadjusted OR, White Australian** = 1.07 [95% CI = 1.02–1.11]^††^  **Unadjusted OR, Chinese Australian** = 1.06 [95% CI = 0.99–1.14] ^††^  **Unadjusted OR, Hong Kong =** 0.97 [95% CI = 0.87–1.08]^††^  **Unadjusted OR, Taiwan =** 1.01 [0.96–1.05]^††^  *^††^Data provided by authors, on request* |
| Landers et al. (2016) | USA, community | 64 | 56 [8] | Mean age = 72.2 yrs ± 7.2  Female = 62.5% | FES (10-100; higher scores = lower falls efficacy/confidence); analysed as a continuous variable  ABC (0-100%); higher scores = greater balance confidence; analysed as a continuous variable | **Inclusion:** Aged ≥60 yrs, community dwelling.  **Exclusion:** Unable to read or speak English, nonadherence, cognitive impairment (Mini-Mental State Examination score <21), or comorbidities that prevented participation in balance testing (e.g., recent surgeries, nonstable medical conditions, painful osteoarthritis with weight bearing, orthostatic hypotension, vestibulopathy) | **Outcome:** Any-type falls and recurrent falls (2+ falls)  **Falls definition:** No falls definition provided  **Method:** Retrospectively assessed at end of follow-up (via telephone) | 1 yr | **Outcome:** Any-type falls (v. no falls)  **FES: Adjusted OR =** 1.00 [95% CI = 0.93–1.07], *p* = .990^††^  **ABC: Adjusted OR =** 0.95 [95% CI = 0.89–1.01], *p* = .081^††^  **Outcome:** Recurrent falls (vs. no falls or single fall)  **FES: Adjusted OR =** 1.07 [95% CI = 0.91–1.24], *p* = .417^††^  **ABC: Adjusted OR =** 0.82 [95% CI = 0.68–1.00], *p* = .048^††^  *^††^Data provided by authors, on request* |
| Lanoue et al. (2020) | Canada, Emergency Department (but community dwelling / not admitted to hospital as in-patient) | 2899 | 2009 [890] | Mean age = 76.2 yrs ± 7.5  Female = 65.6% | Short FES-I (7-28; higher scores = greater concerns about falling); split into mild (7–8), moderate (9–13), and severe concerns (14–28), and also analysed as a continuous variable. | **Inclusion:** Aged ≥65 yrs, independent in all activities of daily living, and presented to emergency department with chief complaints of minor injury sustained in falls (i.e., injury not requiring admission/surgery)  **Exclusion:** Hospitalised patients, and those unable to give consent or to speak French or English. | **Outcome:** Any-type falls  **Falls definition:** “Fall hard enough to feel pain afterwards”  **Method**: Number of falls obtained at 3- and 6-month period after baseline (via telephone and in-person interviews) | 6 mnths | Moderate (vs. mild) concerns:  **Unadjusted OR =** 1.63 [95% CI = 1.21–2.20]  Severe (vs. mild) concerns:  **Unadjusted OR =** 2.37 [95% CI = 1.59–3.52]  Continuous variable:  **Unadjusted OR** = 1.08 [95% CI = 1.05 – 1.12]^††^  *^††^Data provided by authors, on request* |
| Lavedan et al. (2018) | Spain, community | 640 | 395 [245] | Mean age = 81.5 yrs ± 5.0  Female = 60.3% | Single-item question: “Are you afraid of falling?”  Yes/No | **Inclusion:** Aged ≥75 yrs, living at home, coverage by the public health system.  **Exclusion:** Living in residential care, presence terminal illness, or presence of cognitive impairment ( Pfeiffer Short Portable Mental Status Questionnaire > 3) without accompanying carers to aid completion of assessments. | **Outcome:** Any-type falls  **Falls definition:** “The consequence of an event which had resulted in a person inadvertently coming to rest on the ground”  **Method:** Retrospective recall, at end of follow-up period; provided by proxy if suspected cognitive impairment. | 2 yrs | **Unadjusted HR** = 1.93 [95% CI = 1.33–2.81], *p* = .001  **Adjusted HR (Model 1) =** 1.18 [95% CI = 0.79–1.74], *p* = .46  **Adjusted HR (Model 2)** = 1.18 [95% CI = 0.77–1.81], *p* = .43 |
| Lim et al. (2021) | Australia, community | 223 | Full 30-item Icon-FES analysis: 223 [0]  Short 10-item Icon-FES analysis: 108 [115] | Mean age = 79.1 yrs ± = 5.4  Female = 58.8% | Full 30-item IconFES (30-120; higher scores = greater concerns about falling); split into low (30-52) and high (53-120) concerns  Short 10-item IconFES (10-40; higher scores = greater concerns about falling); split into low (10-18) and high (19-40) concerns | **Inclusion:** Aged ≥70 yrs, living independently at home, able to ambulate at home without walking aid, without any acute medical or progressive neurological conditions. | **Outcome:** Recurrent falls (2+ falls) and ‘Serious’ falls (2+ falls and/or 1+ injurious fall)  **Falls definition:** “An unexpected event in which the person comes to rest on the ground, floor, or lower level”  **Method:** Monitored using monthly or weekly falls diaries, and follow-up telephone calls as required | 12 mnths | **Outcome:** Recurrent falls (vs. no falls and single fallers)  **Unadjusted OR (30-item IconFES) =** 1.57 [95% CI = 1.03–2.37], *p* = .034  **Unadjusted OR (10-item IconFES) =** 1.83 [95% CI = 1.16–2.90], *p* = .009  **Outcome:** Serious falls (vs. no falls and single fallers )  **Unadjusted OR (30-item IconFES)** = 1.55 [95% CI = 1.10–2.19], *p* = .012  **Unadjusted OR (10-item IconFES) =** 1.55 [95% CI = 1.05–2.27], *p* = .026 |
| Litwin et al. (2018) | Europe (Austria, Belgium, Czech Republic, Denmark, Estonia, France, Germany, Italy, the Netherlands, Slovenia, Spain, Sweden and Switzerland); community | 22,533 | Unadjust. model= 22,533 [0]  Adjust. Models 1 & 2 = 20,654 [1879]  Adjust. Models 3 & 4 = 19,023 [3510] | Mean age = 74.4 ± 6.9  Female = 56.5% | Single-item question: “For the past six months at least, have you been bothered by a fear of falling down?” Yes/No | *Data collected as part of the Survey of Health, Ageing and Retirement in Europe (SHARE)*  **Inclusion:** Community dwelling Europeans aged 65+ years who participated in both the fourth and fifth waves of SHARE | **Outcome:** Any-type falls  **Falls definition:** No definition provided; participants asked if they had been “bothered by falling down”  **Method:** Asked retrospectively, at end of follow-up period | 2 yrs; but falls only assessed in prior 6 mnths | **Unadjusted OR =** 3.72 [95% CI = 3.38–4.12^††^], *p* < .001  **Adjusted OR (Model 1)** = 1.17 [95% CI = 1.03–1.34^††^], *p* < .05  **Adjusted OR (Model 2)** = 1.71 [95% CI = 1.38–2.16^††^], *p* < .001  **Adjusted OR (Model 3) =** 1.15 [95% CI = 1.04–1.38^††^], *p* < .05  **Adjusted OR (Model 4)** = 1.66 [95% CI = 1.35–2.17^††^], *p* < .001  ^††^95% CIs provided by authors, on request. |
| Luukinen et al. (1996) | Finland, community | 1016 | 979 [37] | Mean age = 76.1 yrs ± 4.9  Female = 63.0% | Single-item question: “Are you afraid of falling?”  Split into ‘frequent’ (answered “frequently” or “always”) vs. ‘non-frequent’ (answered “none” or “sometimes”) | **Inclusion:** Aged ≥70 yrs, community dwelling | **Outcome:** Recurrent falls (2+ falls) vs. no falls or single falls  **Falls definition:** “An unexpected event when the person fell to the ground from any level, including falIs on stairs and falls onto a piece of furniture.”  **Method:** Falls diaries and telephone calls every 3 months | 1 yr | **Unadjusted RR** = 3.00 [95% CI = 2.04–4.39]  **Adjusted OR** = 2.16 [95% CI = 1.27–3.68] |
| Luukinen et al. (1997) | Finland, community | 931 | 790 [141]  Case-controlled design, focusing only on those who experience an injurious during follow-up:  *n* = 82 with a fracture; *n* = 82 with soft-tissue damage | Female = 61.9%  Mean age, females = 76.6 yrs ± 4.9  Mean age, males = 75.5 yrs ± 4.9 | Single-item question: “Are you afraid of falling?”  Split into ‘frequent’ (answered “frequently” or “always”) vs. ‘non-frequent’ (answered “none” or “sometimes”) | **Inclusion:** Community-dwelling, aged ≥70 yrs, who experienced a fall that led to a minor injury, major soft tissue injury, or fracture during the 4 yr follow-up period (case-controlled design)  **Exclusion:** No longer community-dwelling at follow-up | **Outcome:** Falls leading to a fracture vs. falls leading to soft-tissue damage  **Falls definition:** “An unexpected event upon which a person fell to the ground from an upper level or on the same level, including falls on stairs and onto a piece of furniture”  **Method:** Fall diaries were used to record falls and telephone calls every 3-months. Medical records were reviewed at the end of each year | 4 yrs | **Unadjusted OR** = 3.2 [95% CI = 1.55–6.45)  **Adjusted OR =** OR 2.50 [95% CI = 1.11–5.65] |
| Makino et al. (2021) | Japan, community | 4221 | 2151 [2070] | Mean age = 69.3 yrs ± 4.7  Female = 51.6% | Single-item question: “Are you afraid of falling?”  ‘Fear’ (answered “very much” or “somewhat”) vs. ‘No fear’ (answered “a little” or “not at all”) | **Inclusion:** Aged ≥65 yrs, non-faller at baseline, no presence of functional disability, and not participating in any other studies  **Exclusion:** History of either Alzheimer’s disease, stroke, Parkinson’s disease, and/or depression; severe cognitive impairment (Mini-Mental State Examination score of less than 20); presence of a functional disability; not completing the physical frailty, fall, or FOF assessments | **Outcome:** Any-type falls  **Falls definition:** “An unexpected event in which the person comes to rest on the ground, floor, or a lower level.”  **Method:** Retrospective survey, at end of follow-up period | 4 yrs; but falls only assessed in prior 12 mnths | **Adjusted OR =** 1.29 [95% CI = 0.98–1.70], *p* = .069 |
| Marques et al. (2021) | Brazil, community (but participants recruited from ambulatory care) | 121 | 116 [5] | Mean age = 71.1yrs ± 7.4  Female = 69.4% | FES-I (16-64; higher scores = greater concerns about falling); analysed as a continuous variable | **Inclusion:** Aged ≥60 yrs, living in the community, able to ambulate with or without assistive devices, able to stand up independently, and able to understand verbal commands.  **Exclusion:** Cognitive impairment detectable by the Mini-Mental State Examination, neurological and orthopedic sequelae, and cardiorespiratory problems that could prevent gait. | **Outcome:** Any-type falls  **Falls definition:** “Unintentionally coming to the ground or some lower level.”  **Method:** Retrospective phone call, at end of follow-up period | 6 mnths | **Unadjusted OR** = 1.05 [95% CI = 1.00–1.10], *p* = .067^††^  *^††^Data provided by authors, on request* |
| Menant et al. (2016) | Australia, community | 529 | 527 [2] | Mean age = 79.8 yrs ± 4.4  Female = 52.2% | FES-I (16-64; higher scores = greater concerns about falling); analysed as a continuous variable | **Inclusion:** Aged ≥72 yrs, and community-dwelling  **Exclusion:** Mini-Mental State Examination score <24 | **Outcome:** Any-type falls  **Falls definition:** “An unexpected event in which a person comes to rest on the ground, floor, or other lower level”  **Method:** Monthly fall diaries and telephone calls | 1 yr | **Unadjusted OR** = 1.05 [95% CI = 1.02–1.08]^††^  **Adjusted OR** = 1.05 [95% CI = 1.02–1.08]^††^  *^††^Data provided by authors, on request* |
| Moiz et al. (2017) | India, community | 125 | 125 [0] | Mean age = 70.2 yrs ± 6.39  Female = 29.6% | ABC (0-100%; higher scores = greater balance confidence); Used as both a continuous and dichotomous high (59-100) and low (0-58) confidence | **Inclusion:** Age ≥60 yrs, and could read and communicate in Hindi  **Exclusion:** Those who received physiotherapy regimen or those with a history of psychotic and/or cognitive problems | **Outcome:** Any-type falls  **Falls definition:** “Any event when the resident unintentionally comes to rest on the floor, regardless of the cause”  **Method:** Monthly mailed fall calendars printed on post-cards. | 1 yr | ABC, continuous:  **Unadjusted OR** = 0.83 [95% CI = 0.77–0.90], *p* < .001  **Adjusted OR (Model 1)** = 0.84 [95% CI = 0.75–0.96], *p =* 0.009  ABC, dichotomous:  **Unadjusted OR** = 0.02 [95% CI = 0.00–0.08], *p <* 0.001  **Adjusted OR (Model 1) =** 0.032 [95% CI = 0.00–0.25], *p* = 0.001 |
| Okoye et al. (2023) | USA, community | 6489 | 5093 [1396]  *Note: 1396 included those who did not complete follow-up test, or who had probable dementia.* | 65-74 yrs = 60.1%; 75-84 yrs = 30.8%; 85+ yrs = 9.1%  Female = 55.9% | Single-item question: “Have you worried about falling in the past month?”  Yes/No | *Data obtained from Round 5 (2015) and Round 6 (2016) of the National Health and Aging Trends Study (NHATS)*  **Inclusion:** Aged ≥65 yrs, community dwelling.  **Exclusion:** Probable dementia; lived in residential care facilities or nursing homes; did not provide information about home environment. | **Outcome:** Any-type falls  **Falls definition:** “Any fall, slip, or trip in which you lose your balance and land on the floor or ground or at a lower level.”  **Method:** In-person interview (home visit) at follow-up | 1 yr | **Unadjusted OR =** 2.61 [95% CI = 2.17–3.14**]**  **Adjusted OR** = 1.65 [95% CI = 1.34–2.02] |
| Pereira et al. (2021) | Portugal; community | 513 | 280 [233] | Female = 77.8%  Mean age, females = 73.2 yrs ± 5.6  Mean age, males = 74.0 yrs ± 6.1 | FES-I (16-64; higher scores = greater concerns about falling); analysed as a continuous variable. | **Inclusion:** Aged ≥65 yrs, independent mobility, absence of recent injuries that have caused temporary immobilization, deafness or blindness, and absence of severe cognitive impairment (Mini-Mental State Examination ≥9). | **Outcome:** Any-type falls  **Falls definition:** “An unexpected event in which the participants come to rest on the ground, floor, or lower level”  **Method:** Telephone calls at 6 and 12 months | 1 yr | **Unadjusted OR** = 1.04 [95% CI = 1.01–1.08]^††^  **Adjusted OR** = 1.02 [95% CI = 0.98–1.06]^††^  *^††^Data provided by authors, on request* |
| Pluijm et al. (2006) | Netherlands; community | 1365 | 1246 [119] | Mean age = 75.3 yrs ± 6.4  Female = 51.1% | FES, modified version (0–30; higher scores = greater concerns about falling, rather than falls efficacy/ confidence)  Participants split into absence (0/30) vs. presence (≥1/30) of concerns | *Data collected as a subsample of the Longitudinal Aging Study Amsterdam (LASA) cohort.*  **Inclusion: P**articipated in the second data collection cycle of LASA (1995/1996), aged ≥65 yrs, and living in the community. | **Outcome:** Recurrent falls (2+ falls within any 6-month period during 3-year follow-up) vs. no falls and single falls  **Fall definition:** “An unintentional change in position resulting in coming to rest at a lower level or on the ground”  **Method:** Falls recorded weekly on a calendar (mailed to participants) | 3 yrs | **Unadjusted OR** = 1.90 [95% CI = 1.45–2.49]  **Adjusted OR** = 1.40 [95% CI = 1.01–1.93] |
| Porto et al. (2020) | Brazil; community | 105 | 101 [4] | Mean age = 67.6 yrs ± 5.0  Female = 77.2% | Single item-question: “Are you afraid of falling?”  Yes/No | **Inclusion:** Independent and autonomous community-dwelling older adults aged ≥60 yrs.  **Exclusion:** A history of fall during the 12 months preceding the initial evaluation; musculoskeletal or neurological conditions that could interfere with performance in the functional tests or increase the risk of falls by themselves (daily pain, prostheses, recent or not consolidated fractures, symptomatic orthopedic dysfunctions of the spine and lower limbs such as osteoarthritis and tendinitis, Parkinson´s disease or motor sequelae of a stroke); dizziness; visual complaints that would jeopardize the execution of daily activities (self-report); deficit of foot protecting sensitivity; cardiovascular or metabolic conditions that would contraindicate physical activities, and a low score on the 10-point Cognitive Screener according to educational level (< 8 points) | **Outcome**: Any-type falls  **Falls definition**: “An unintentional event resulting in a change of the participant’s position to a lower level than the initial position”  **Method:** Monthly telephone contact | 1 yr | **Unadjusted OR** = 0.66 [95% CI = 0.27–1.59], *p*=.364  **Adjusted OR** = 0.85 [95% CI = 0.32–2.25], *p*=.754 |
| Roman de Mettelinge & Cambier (2015) | Belgium, residential aged care | 43 | 42 [1] | Mean age = 83.2 yrs ± 7.1  Female = 74.4% | Single-item question: “Are you afraid of falling?”  Split into Fear (‘slightly afraid’, ‘somewhat afraid’, or ‘very afraid’) vs. No Fear (‘not at all afraid’) | **Inclusion:** Aged ≥60 yrs; able to walk independently for 10+ m; absence of neurological disorders | **Outcome:** Any-type falls  **Falls definition:** “an unexpected event in which the person comes to rest on the ground, floor, or lower level”  **Method:** Monthly fall calendars and telephone calls in case a fall occurred | 1 yr | **Unadjusted OR =** 1.44 [95% CI = 0.39–5.34] |
| Svoboda et al. 2017 | Czech Republic, community | 125 | 125 [0] | Mean age = 70.6 yrs ± 6.5  Female = 80.8% | FES-I (16-64; higher scores = greater concerns about falling)  ABC (0-100%; higher scores = higher balance confidence)    Both analysed as a continuous variable | **Inclusion:** Aged ≥60 yrs, ability to walk without an assistive device, and the ability to stand unassisted without any support during common everyday activities  **Exclusion:** Neurological or vestibular disease and surgery in lower limbs or spine during the last two years | **Outcome:** Any-type falls  **Falls definition:** “An unexpected event in which the participants come to rest on the ground, floor, or lower level”  **Method:** Every two weeks, via telephone. | 6 mnths | FES-I:  **Unadjusted OR =** 1.02 [95% CI = 0.94–1.10), *p* = .67^††^  ABC:  **Unadjusted OR =** 0.99 [95% CI = 0.96–1.03), *p* = .69^††^  *^††^Results from open-access data provided in the original paper.*  *Note: As non-significant in univariate model, not entered into full (adjusted) model.* |
| Trevisan et al. (2020) | Italy, community & nursing homes | 3099 | 2097 [1002] | Mean age = 75.4 yrs ± 7.3  Female = 58.9% | Single-item question: “Afraid of falling?”  Yes/No | **Inclusion:** Aged ≥65 yrs, residing in one of two cities in Northern Italy (either in the community or nursing home).  **Exclusion:** No exclusion was used | **Outcome:** Any-type falls and recurrent falls (2 or more falls)  **Falls definition**: ‘‘An unexpected event where a person falls to the ground from an upper level or the same level’’  **Method:** Trained nurses and physicians assessed the study participants at baseline (between 1995 and 1997), and made follow-up assessments after about 4 years. At the follow-up assessments, trained nurses recorded the number of accidental falls in the previous year in face-to-face interviews with participants or with their caregivers. | Mean follow-up period: 4.4 yrs | **Outcome:** Any-type falls (vs. no falls)  **Adjusted OR (Model 1) =** 1.25 [95% CI = 1.18–1.33]  **Adjusted OR (Model 2)** = 1.07 [95% CI = 1.00–1.14]  **Adjusted OR (Model 3)** = 1.04 [95% CI = 0.97–1.11]  **Outcome:** Recurrent falls (vs. no falls)  **Adjusted OR (Model 1) =** 2.27 [95% CI = 2.08–2.47]  **Adjusted OR (Model 2)** = 1.99 [95% CI = 1.81–2.18]  **Adjusted OR (Model 3)** = 1.87 [95% CI = 1.70–2.05] |
| Tromp et al. (2001) | Netherlands; community | 1374 | 1285 [89] | Mean age = 75.2 yrs ± 6.5  Female = 51.1% | FES, modified version (0–30; higher scores = greater concerns about falling, rather than falls efficacy/ confidence)  Participants split into absence (0/30) vs. presence (≥3/30) of concerns. | *Data collected as a subsample of the Longitudinal Aging Study Amsterdam (LASA) cohort.*  **Inclusion:** Participated in the second data collection cycle of LASA (1995/1996), aged ≥65 yrs, and living in the community. | **Outcome:** Any-type falls and recurrent falls (2+ falls)  **Fall definition:** “An unintentional change in position resulting in coming to rest at a lower level or on the ground”  **Method:** Falls recorded weekly on a calendar (mailed to participants); contacted every 3 months by telephone if no falls calendar returned, or if errors were made on returned calendars. | 1 yr | **Outcome:** Any-type falls (vs. no falls)  **Unadjusted OR** = 1.80 [95% CI = 1.30–2.30]  **Outcome:** Recurrent falls (vs. no falls or single falls)  **Unadjusted OR** = 2.00- [95% CI = 1.40–2.80] |
| Tsang et al. (2022) | Hong Kong, community | 480 | 461 [19] | Mean age = 70.6 yrs ± 7.1  Female = 81.0% | ABC (0-100%; higher scores = greater balance confidence); split into high (≥76/100) vs low confidence, and analysed as a continuous variable. | **Inclusion:** Aged ≥60 yrs, community-dwelling, ≥ 1 fall in the past year, able to understand Chinese and able to walk for ≥ 10 meters. The use of orthosis and/or walking aids was allowed.  **Exclusion:** Uncorrectable visual impairment. | **Outcome**: Any-type falls (1+ fall) and injurious falls (‘any fall resulting in an injury’)  **Falls definition:** “Unintentionally coming to rest on the ground, floor or other lower level”  **Method:** Tri-monthly telephone follow-ups | 1 yr | **Outcome:** Any-type falls (vs. no falls)  High vs low confidence:  **Unadjusted OR** = 0.40 [95% CI = 0.25–0.63], *p*<.001  **Adjusted OR** = 0.52 [95% CI = 0.32–0.86], *p*=.010  Continuous variable:  **Unadjusted OR** = 0.98 [95% CI = 0.97–0.99], *p* = .002^††^  **Adjusted OR** = 0.98 [95% CI = 0.97–0.99], *p* = .043^††^  **Outcome:** Injurious falls (vs. anyone else)  High vs low confidence:  **Adjusted OR** = 0.57 [95% CI = 0.33–0.97], *p*=.037  Continuous variable:  **Adjusted OR =** 0.99 [95% CI = 0.97–1.00], *p* = .095^††^  *^††^Data provided by authors, on request* |
| van Gulick et al. (2022) | Netherlands, community | 425 | 407 [18] | Mean age = 74.3 yrs ± 5.8  Female = 60.4% | Single-item question: “Are you afraid of falling?”  Yes/No | **Inclusion:** Aged ≥65 years, living in the community, and indicated for a podiatric consultation.  **Exclusion:** Patients who were unable to walk 6 m, could not stand unassisted for 30 s without shoes, had insufficient command of Dutch or English, or had severe cognitive impairments precluding reliable (self-report) data collection at baseline or follow-up were excluded | **Outcome:** Any-type falls  **Falls definition**: “An unexpected event in which the participant comes to rest on the ground, floor, or lower level”  **Method**: Falls calendar returned at the end of each quarter, and follow-up telephone call | 1 yr | **Adjusted OR** = 1.25 [95% CI = 0.76–2.06^††^], *p* = .377  *^††^Cis provided by authors, on request* |
| van Schooten et al. (2015) | Netherlands, community and residential aged care | 169 | 169 [0] | Mean age = 75.4 yrs ± 6.8  Female = 52.1% | FES-I (16-64; higher scores = greater concerns about falling); analysed as continuous variable | **Inclusion:** Aged 65-99 yrs, had a Mini-Mental State Examination score of ≥19, and were able to walk at least 20 meters with aid of an assistive device if needed | **Outcome:** Any-type falls  **Falls definition:** “Events that resulted in a person coming to rest unintentionally on the ground or other lower level”  **Method:** Monthly telephone contact in addition to fall diaries to be filled out daily. | 6 mnths | **Unadjusted OR** = 1.06 [95% CI = 1.00–1.12], *p*<0.05 |
| van Schooten et al. (2021) | Australia, community | 500 | 494 [6] | Mean age = 78.0 yrs ± 4.6  Female = 54.0% | FES-I (16-64; higher scores = greater concerns about falling); analysed as a dichotomous variable for IRR analysis (participants split into high ( ≥23/64) and low (<23/64) concerns) and a continuous variable for OR analysis | **Inclusion:** Aged between 70-90 yrs, neurologically in-tact.  **Exclusion:** Diagnosis of dementia, psychotic symptoms, or a diagnosis of progressive (inc. Parkinson’s Disease and Multiple Sclerosis) or unstable medical conditions. | **Outcome:** Any-type falls  **Falls definition:** “Events that resulted in a person coming to rest unintentionally on the ground or other lower level”  **Method:** Monthly fall calendars; and follow-up telephone call if not returned. | 1 yr | **Adjusted IRR (Model 1) =** 1.68 [95% CI = 1.25–2.25]  **Adjusted IRR (Model 2)** = 1.61 [95% CI = 1.19–2.18]  **Adjusted OR (Model 1)** = 1.04 [95% CI = 1.01–1.07]^††^  **Adjusted OR (Model 2)** = 1.04 [95% CI = 1.00–1.07]^††^  *^††^Data provided by authors, on request* |
| Ward et al. (2015) | USA, community | 765 | 755 [10] | Mean age = 78.1 yrs ± 5.4  Female = 64.1% | FES (1-10; higher scores = greater falls efficacy/ confidence); analysed as a continuous variable (with HRs presented per standard deviation) | **Inclusion:** Aged ≥70 yrs, ability to walk 20 feet without the aid of another person, and intention to stay in the Boston area for 2 years or longer.  **Exclusion:** Moderate to severe cognitive impairment (Mini-Mental State Examination score <18), severe visual or hearing deficits, and terminal illness. | **Outcome:** Incidence of injurious falls (falls leading to fractures; sprains; dislocations; pulled or torn muscles, ligaments, or tendons; or to medical attention)  **Falls definition: “**Unintentionally coming to rest on the ground or another lower level not resulting from a major health event (e.g., myocardial infarction) or an overwhelming external hazard (e.g., vehicular accident).”  **Method:** Daily falls calendars. Associated injuries were ascertained through structured interviews. | 4 yrs | **Adjusted HR (Model 1)** = 0.90 [95% CI = 0.80–1.02]  **Adjusted HR (Model 2)** = 0.92 [95% CI = 0.81–1.04] |
| Weijer et al. (2018) | Netherlands, community and residential home | 416 | 272 [144] | Mean age = 75.2 yrs ± 6.9  Female = 50.7% | FES-I (16-64; higher scores = greater concerns about falling); participants split into high (60-100^th^ percentile) and low (0-40^th^ percentile), and analysed as a continuous variable. | **Inclusion:** Aged ≥65 yrs, MMSE score ≥19/30, able to walk 20+m (with walking aid if needed) | **Outcome:** Any-type falls  **Falls definition:** “An unintentional change in position resulting in coming to rest at a lower level or on the ground”  **Method:** Fall diary and monthly telephone call | 6 mnths | High vs. low FES-I:  **Adjusted OR =** 1.03 [90% CI = 0.60–1.75]  Continuous variable analysis:  **Adjusted OR** = 1.05 [95% CI = 1.00–1.09]^††^  *^††^Data provided by authors, on request* |
| Weijer et al. (2021) | Netherlands, Community | 118 | 118 [0]  *Note: This was a subsample of n=287 from a previous sample (who had not fallen at baseline).* | Mean age = 71.4 yrs ± 5.3  Female = 69.5% | FES-I (16-64; higher scores = greater concerns about falling); analysed as a continuous variable | **Inclusion:** Aged ≥65+ yrs, Mini-Mental State Examination score >19/30, able to walk at least 20 m (with walking aid if needed, without becoming short of breath or suffering chest pain), no falls in previous year.  **Exclusion:** One or more retrospective falls | **Outcome**: Any-type falls and injurious falls  **Falls definition**: “An unintentional change in position resulting in coming to rest at a lower level or on the ground”  **Method**: Falls diary and monthly telephone call. | 1 yr | Any-type falls:  **Adjusted OR =** 1.08, [95% CI = 0.97–1.22], *p* = .173  Injurious falls (vs. non-fallers):  **Adjusted OR =** OR = 1.07 [95% CI = 0.97–1.20], *p* = .177 |
| Welmer et al. (2023) | Sweden, community | 1366 | 1281 [85] | Mean age = 72.4 yrs ± 12.8  Female = 62.5% | Single-item question:  Winter FOF: “Are you afraid of falling when you go outdoors in winter?” Yes/No  Other-seasons FOF**:** “Are you afraid of falling when you go outdoors in seasons other than winter?” Yes/No | *Data drawn from the population-based Swedish National Study on Aging and Care in Kungsholmen (SNAC-K).*  **Inclusion:** Aged ≥60 yrs, living in central Stockholm.  **Exclusion:** Living in a nursing home, cognitive impairment (Mini-Mental State Examination score <24) | **Outcome:** Incidence of injurious fall  **Falls definition:** “A fall causing an injury that required inpatient or outpatient care” (other than falling from height)  **Method:** Data obtained via hospital/medical records | Until first injurious fall, death, or the end of the follow-up period (up to 5 years). | Winter FOF:  **Adjusted HR (Model 1) =** 1.47 [95% CI = 1.15–1.91)  **Adjusted HR (Model 2)** = 1.42 [95% CI = 1.10–1.83)  **Adjusted HR (Model 3)** = 1.21 (95% CI = 0.94–1.59)  FOF in other seasons that Winter:  **Adjusted HR (Model 3) =** 0.94 [95% CI = 0.70–1.27] |
| Wijlhuizen et al. (2007) | Netherlands, community | 2080 | 1752 [328] | Mean age = 73.0 yrs ± 5.8  Female = 58.0% | Single item question: “how often afraid of falling outdoors?”  Low (‘never’ or ‘seldom’) vs. High (‘regular’ or ‘very often’) | **Inclusion:** Aged ≥65 yrs, living in their own homes | **Outcome**: Outdoor falls (at least one fall outdoors during walking or bicycling)  **Falls definition:** A fall outdoors during walking or bicycling  **Method**: Telephone call once a month. | 10 mnths | **Adjusted OR (Model 1) =** 1.70 [95% CI = 0.90–3.20], *p*=.10  **Adjusted OR (Model 2)** = 2.00 [95% CI = 1.10–3.90], *p*=.03 |
| Yang & Pepper (2020) | USA, community | 47 | 47 [0] | Mean age = 78.9 yrs ± 5.5  Female = 74.5% | M-FES (0-100%, with 20% indicators along the scale; higher scores = greater falls efficacy/ confidence); participants split into High (≥86/100) and Low falls efficacy (<86/100) | **Inclusion:** Aged ≥70 yrs, community-dwelling, able to read and speak English, able to stand unsupported for 30s, have access to a telephone, and taking drugs from one or more of the following drugs associated with falls at a stable dose for at least 2 months: antipsychotics, antidepressants, benzodiazepine, sedative/hypnotics, type 1A antiarrhythmics, digoxin, diuretics, analgesics, antihypertensives, or agents with anticholinergic properties.  **Exclusion:** diagnosed neuromotor or vestibular disease, severely impaired vision (<20/200) by Snellen chart when using corrective lenses, self‐report of severely impaired range of motion at the hip, knee, or ankle, self‐report of severe kinesthesia of the toes or ankles, cognitive impairment evidenced by Mini-Mental State Examination <24, or any disorders, illnesses, or injuries that the participant judged might interfere significantly with measurement of balance and other activities required in the study | **Outcome**: Incidence of fall events (both actual falls and near falls)  **Falls definition**: “Actual falls were defined as unintentionally coming to rest on the ground, floor, or other lower level. Near falls occurred when participants felt falls were imminent but were avoided by a compensatory action”  **Method:** Falls diary to record fall data, and postcards for monthly report of fall events and fall event-related outcomes. If a postcard was not received for a month, telephone contact was initiated. | 1 yr | **Adjusted IRR (Model 1)** = 0.97 [95% CI = 0.93–1.00]  **Adjusted IRR (Model 2)** = 0.96 [95% CI = 0.92–0.99] |

^a^ Please see Supplementary Appendix C for a full list of the covariates controlled for in all adjusted analyses presented.

ABC = Activities Balance Confidence scale

CI = Confidence Interval

FES = Falls Efficacy Scale

FES-I = Falls Efficacy Scale International

FOF = Fear of falls

HR = Hazards Ratio

IRR = Incidence Rate Ratio

OR = Odds Ratio

**Appendix C.** **Adjusted covariates in included studies**

| **Study (author and year)** | **Adjusted variables** |
| --- | --- |
| Allali et al. (2017) | **FOF:** Age, sex, education, postural instability/gait difficulty (PIGD), bradykinesia, rigidity, global health score (GHS), falls in the past 12 months, gait velocity, Geriatric Depression Scale-15 (GDS-15), and Repeatable Battery for the Assessment of Neuropsychological Status (RBANS).  **ABC:** Age, sex, education, PIGD, bradykinesia, rigidity, GHS, falls in the past 12 months, gait velocity, GDS-15, and RBANS total score. |
| Aoyama et al. (2010) | No adjusted analyses presented |
| Asai et al. (2022) | Age, sex, Timed up and Go, and polypharmacy |
| Burns et al. (2022) | No adjusted analyses presented |
| Cleary & Skornyakov (2017) | No adjusted analyses presented |
| Clemson et al. (2015) | No adjusted HRs presented, as the significance level for FOF was above the cut-off required to enter the variable into the full model (p = .010); and thus, FOF was not entered into the adjusted model. |
| Crenshaw et al. (2020) | No adjusted analyses presented |
| Cumming et al. (2000) | Age, sex, falls in past year (0–5), activities of daily living score (0–10), use of walking aid, history of stroke, use of psychotropic medications, impaired vision, and randomization group. |
| Delbaere et al. (2004) | Age, sex and fear-related activity restriction of activities of daily living. |
| Delbaere et al. (2006) | No adjusted analyses presented |
| Delbaere et al. (2010) | Physiological fall risk (physiological profile assessment) |
| Duan et al. (2022) | Age, 2 min step test, 8ft up-and-go test |
| Faulkner et al. (2009) | Model 1 (‘basic’ model): age, fall history at baseline, and recruitment clinic.  Model 2 (fully adjusted model): height, dizziness, visual acuity, self-rated health, fall history at baseline, use of benzodiazepines, use of antidepressants, use of antiepileptics, difficulty with instrumental activities of daily living, standing balance eyes closed, walking speed, smoker, physical activity, frequency going outdoors, age, recruitment clinic, waist-to-hip circumference, stroke, Parkinson's disease, diabetes, arthritis, self-rated health, standing balance with eyes open, rapid stepping, grip strength, alcohol consumption, hours per day spent on feet, and hours per week does household chores. |
| Friedman et al. (2002) | Sex, history of stroke, Parkinson’s disease, comorbidity index, ethnicity, falls at baseline, General Health Questionnaire score, age, medication 4 or more. |
| Gade et al. (2021) | No adjusted analyses presented |
| Garbin et al. (2023) | Balance, age, sex, race, number of comorbidities, and fall history |
| Gasmann et al. (2009) | No adjusted analyses presented |
| Hadjistavropoulos et al. (2007) | Sex, age, pain severity, Behavioral Rating Scale, medical risk factors, pain-related fear/anxiety subscales of the Pain Anxiety Symptoms Scale (PASS), FES, ABC, fear-of-falling subscale of the Survey of Activities and Fear of Falling in the Elderly (SAFFE), escape-avoidance subscale of the PASS, and the activity level and restriction subscales of the SAFFE |
| Helsel et al. (2021) | No adjusted analyses presented |
| Hicks et al. (2020) | Age, sex, previous falls, Timed up and Go, anxiety (Generalised Anxiety Disorder Questionnaire (GAD-7)). |
| Kamide et al. (2019) | Model 1: Age and sex  Model 2: Age, sex, and Timed up and Go |
| Kamide et al. (2021) | Age, sex, BMI, fall history and depressive symptoms and grip-strength |
| Kwan et al. (2013) | Model 1: Age, sex, follow-up period  Model 2: Age, sex, cohort, education, incontinence, Parkinson’s Disease |
| Landers et al. (2016) | Faller status: FES, ABC, age, sex, previous falls, Timed up and Go  Frequent faller status: FES, ABC, age, sex, previous falls, Timed up and Go, fear-related activity avoidance |
| Lanoue et al. (2020) | No adjusted analyses presented |
| Lavedan et al. (2018) | Model 1: Age and sex  Model 2: Age, sex, comorbidity, cognitive impairment, symptoms of depression, disability, risk of malnutrition, previous falls |
| Lim et al. (2021) | No adjusted analyses presented |
| Litwin et al. (2018) | Model 1: Age, sex, marital status, education, country, cognition (memory, numeracy, fluency), depressive symptoms, BMI, eyesight, hearing, medication, comorbidity, and previous falls  Model 2: same as Model 1, with additional adjustment for the interaction of baseline fear of falling and mobility limitation at follow-up  Model 3: same as Model 1, with additional adjustment of frailty  Model 4: same as Model 3, with additional adjustment for the interaction of baseline fear of falling and mobility limitation at follow-up |
| Luukinen et al. (1996) | Age, sex, urinary urgency, a poor pulse rise after standing up, dizziness, urinary incontinence, previous fall in past 12 months |
| Luukinen et al. (1997) | Knee strength, visual acuity, and social participation rate |
| Makino et al. (2021) | Frailty status, age, sex, hypertension, diabetes mellitus, heart disease, pulmonary disease, knee osteoarthritis, prescribed medication, pain, cognition (MMSE), depression (GDS) |
| Marques et al. (2021) | No adjusted analyses presented, as FES-I was not significant in univariable model |
| Menant et al. (2016) | Age and sex (provided upon request) |
| Moiz et al. (2017) | Age, sex, BMI, number of comorbidities, number of medications, any fall in previous year, and two prior falls in the previous year |
| Okoye et al. (2023) | Age, sex, education, race, income, financial hardship, history of falling, poor lower extremity performance, visual impairment, hearing impairment, depressive symptoms, physical activity, self-care disability, living arrangement, quality of home, disrepair of community in which they live, social deprivation, living in city vs. non-city. |
| Pereira et al. (2021) | Age, sex, depression (GDS), balance (Fullerton Advanced Balance scale) |
| Pluijm et al. (2006) | Two falls in the previous year, dizziness, functional limitations (≥3), grip strength (women ≤ 32 kg; men ≤56 kg), body weight (women ≤ 62 kg; men ≤70 kg), dogs or cats in household, education ≥11 year, alcohol use (≥18 consumptions per week), and interaction terms (Alcohol use × education; ≥Two falls in the previous year × FOF) |
| Porto et al. (2020) | Age, sex, BMI |
| Roman de Mettelinge & Cambier (2015) | No adjusted analyses presented |
| de Souza et al. (2019) | Age, sex, perceived health, number of diseases, number of medications, hospitalization in the past year, Short Physical Performance Battery (SPPB); dependence for basic activities of daily living; dependence for instrumental activities of daily living. |
| Svoboda et al. (2017) | No adjusted analyses presented, as FES-I was not retained in multivariable model. |
| Trevisan et al. (2020) | Model 1: Age, sex, education, and previous falls.  Model 2: Model 1, but additionally adjusted for living alone, physical activity, vision impairment, diabetes, lower limb osteoarthritis, BMI, depression (Geriatric Depression Scale), and cognition (Mini Mental State Examination).  Model 3: Model 2, but additionally adjusted for Short Physical Performance Battery. |
| Tromp et al. (2001) | No adjusted analyses presented, as the fear of falling variable was not retained in the multivariable regression. |
| Tsang et al. (2022) | Physiological fall risk (physiological profile assessment), recurrent faller (past year), gait speed, history of depression, comorbidities, sex, walking aid |
| van Gulick et al. (2022) | Fall history in the previous year, feeling unsteady while standing and walking, use of a walking aid. |
| van Schooten et al. (2015) | No adjusted analyses presented, as FES-I was not retained in multivariable model. |
| van Schooten et al. (2021) | Model 1: Age, sex, BMI, and cognition (Mini Mental State Examination (MMSE)).  Model 2: Age, sex, BMI, MMSE, executive impairment (dichotomous), Physiological fall risk (physiological profile assessment), and depressive symptoms (dichotomous). |
| Ward et al. (2015) | Model 1: Age, sex, race, psychotropic medication use, and depression  Model 2: Model 1, with addition of Short Physical Performance Battery |
| Weijer et al. (2018) | Age, sex, body weight and body height, symptoms of depression (GDS), executive functioning (trail making test), and fall history. |
| Weijer et al. (2021) | Age, sex and average total walking duration per day (as determined from one-week inertial sensor monitoring) |
| Welmer et al. 2023 | Model 1: age, sex, education (elementary school, high school, or university and above)  Model 2: Model 1, with previous injurious falls  Model 3: Model 2, with balance impairment (ability to balance on one leg for 5 seconds or longer), cohabitation status, instrumental activities of daily living dependence, and the number of drugs. |
| Wijlhuizen et al. (2007) | Model 1: Age and sex  Model 2: Age, sex, and outdoor physical activity level |
| Yang & Pepper (2020) | Model 1: Fall history  Model 2: Age, sex, and fall history |

**Appendix D.** **Proportion and/or rate/number of falls in each study.**

| **Study (author and year)** | **Fallers, number (and percentage)** |
| --- | --- |
| Allali et al. (2017) | **Outcome:** Any-type falls = 169/449 (37.6%) |
| Aoyama et al. (2010) | **Outcome:** Any-type falls = 25/58 (43.1%) |
| Asai et al. (2022) | **Outcome:** Any-type falls = 97/530 (18.3%) |
| Burns et al. (2022) | **Outcome:** Any-type falls = 603/1563 (38.6%) |
| Cleary & Skornyakov (2017) | **Outcome:** Any-type falls = 11/45 (24.4%) |
| Clemson et al. (2015) | **Outcome:** Injurious falls = 200/904 (22.1%) |
| Crenshaw et al. (2020) | **Outcome:** Any-type falls = 74/125 (59.2%) |
| Cumming et al. (2000) | **Outcome:** Any-type falls = 216/528 (41.0%) |
| de Souza et al. (2019) | **Outcome:** Single falls = 59/345 (17.1%)  **Outcome:** Recurrent falls = 69/345 (20.0%) |
| Delbaere et al. (2004) | **Outcome:** Any-type falls = Data not reported.  **Outcome:** Recurrent falls = 47/221 (21.3%) |
| Delbaere et al. (2006) | **Outcome:** Recurrent falls = 52/257 (20.2%) |
| Delbaere et al. (2010) | **Outcome:** Any-type falls = 214/494 (43.3%)  **Outcome:** Serious falls = 166/494 (33.6%) |
| Duan et al. (2022) | **Outcome:** Any-type falls = 35/299 (11.7%) |
| Faulkner et al. (2009) | **Outcome:** Any-type falls = 4995/8378 (59.6%) |
| Friedman et al. (2002) | Data not reported. |
| Gade et al. (2021) | **Outcome:** Any-type falls = 87/198 (43.9%) |
| Garbin et al. (2021) | **Outcome:** Any-type falls = 1682/5151 (32.7%) |
| Gasmann et al. (2009) | **Outcome:**  Any-type falls = 107/622 (17.2%)  Single fall = 71/622 (11.4%)  Recurrent falls = 36/622 (5.8%) |
| Hadjistavropoulos 2007 | **Outcome:** Any-type falls = 128/492 (26.0%) |
| Helsel 2021 | Data not reported. |
| Hicks 2020 | **Outcome:** Any-type falls = 130/313 (41.5%) |
| Kamide 2019 | **Outcome:** Any-type falls = 42/237 (17.7%) |
| Kamide 2021 | **Outcome:** Any-type falls = 25/204 (12.3%) |
| Kwan 2013 | **Outcome:** Any-type falls = 485/1436 (37.8%)  **Outcome:** Recurrent falls = 185/1436 (12.9%)  *Note, this data was provided by the authors upon request.* |
| Landers 2016 | **Outcome:**  Any-type falls = 18/56 (32.1%)  Recurrent falls = 9/56 (16.1%) |
| Lanoue 2020 | **Outcome:** Any-type falls = 250/2009 (12.4%) |
| Lavedan 2018 | **Outcome:** Any-type falls = 11.6% of males and 50.4% of females. *Not possible to calculate the overall numbers/percentage, as the paper does not state how many males and females were included at follow-up.* |
| Lim 2021 | **Outcome:**  Recurrent falls = 42/223 (18.8%)  Serious falls = 76/223 (34.1%) |
| Litwin 2018 | **Outcome:** Any-type falls = 2422/22533 (10.8%) |
| Luukinen 1996 | **Outcome:** Any-type falls = 88/979 (9.0%) |
| Luukinen 1997 | **Outcome:** Case controlled design, falls leading to a fracture vs. falls leading to soft-tissue damage. *N* = 82 in both groups. |
| Makino 2021 | **Outcome:** Any-type falls = 292/2151 (13.6%) |
| Marques 2021 | **Outcome:** Any-type falls = 27/116 (23.3%) |
| Menant 2016 | **Outcome:** Any-type falls = 237/527 (45.0%)  *Note, this data was provided by the authors upon request.* |
| Moiz 2017 | **Outcome:** Any-type falls = 22/125 (17.6%) |
| Okoye et al. (2022) | **Outcome:** Any-type falls = 1574/5093 (30.9%) |
| Pereira et al. (2021) | **Outcome:** Any-type falls = 220/508 (43.3%) |
| Pluijm et al. (2006) | **Outcome:** Recurrent falls = 337/1365 (24.7%) |
| Porto et al. (2020) | **Outcome:** Any-type falls = 29/101 (28.7%) |
| Roman de Mettelinge & Cambier (2015) | **Outcome:** Any-type falls = 20/42 (47.6%) |
| Svoboda et al. (2017) | **Outcome:** Any-type falls = 30/124 (24.2%) |
| Trevisan et al. (2020) | **Outcome:**  Any-type falls = 774/2097 (36.9%)  Recurrent falls = 310/2097 (14.8%) |
| Tromp et al. (2001) | **Outcome:**  Any-type falls = 457/1285 (33.3%)  Single falls = 300/1285 (21.9%)  Recurrent falls = 156/1285 (11.4%) |
| Tsang et al. (2022) | **Outcome:**  Any-type falls = 108/461 (23.4%)  Injurious falls = 87/461 (18.9%) |
| van Gulick et al. (2022) | **Outcome:** Any-type falls = 136/407 (33.4%) |
| van Schooten et al. (2015) | **Outcome:** Any-type falls = 59/169 (34.9%) |
| van Schooten et al. (2021) | **Outcome:** Any-type falls = 214/494 (43.3%)  *Note, this data was provided by the authors upon request.* |
| Ward et al. (2015) | **Outcome:** Injurious falls = 221/775 (29.3%) |
| Weijer et al. (2018) | **Outcome:** Any-type falls = 91/272 (33.5%) |
| Weijer et al. (2021) | **Outcome:**  Any-type falls = 60/118 (50.9%)  Injurious falls **=** 40/118 (33.9%) |
| Welmer et al. 2023 | **Outcome:** Injurious falls = 272/1281 (22.2%) |
| Wijlhuizen et al. (2007) | **Outcome:** Outdoor falls = 52/1752 (3.0%) |
| Yang & Pepper (2020) | **Outcome:** Any-type falls = 34/47 (72.3%) |

**Appendix E. Funnel plot of meta-analyses.**

**Figure E1. Funnel plot for full 16-item Falls Efficacy Scale International (FES-I) and future any-type falls.**


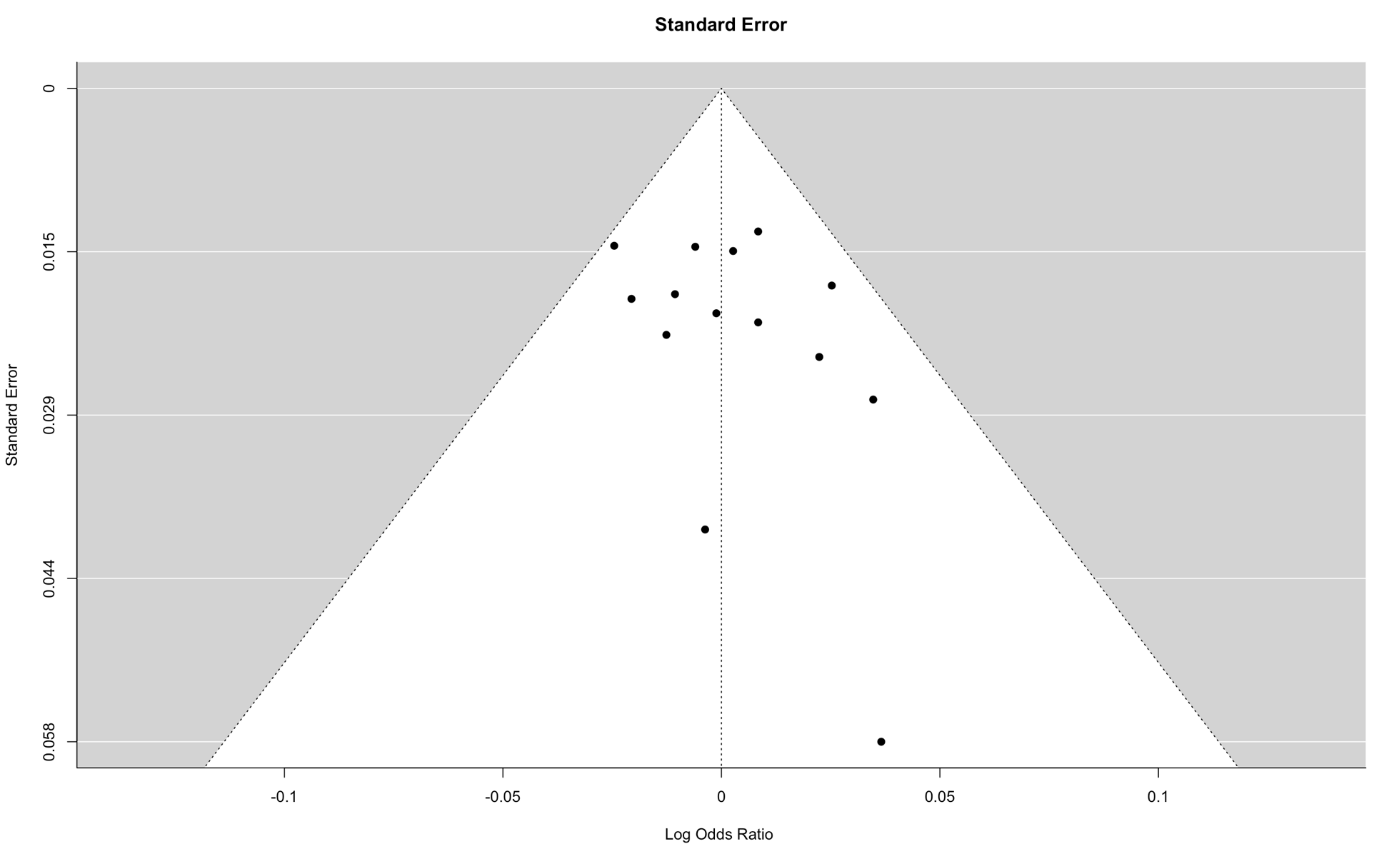


Both visual inspection for asymmetry and the results of Egger’s linear regression test (Z = 0.99, *P* = 0.32) suggest a lack of publication bias.

**Figure E2. Funnel plot for single-item measures of concerns about falling and future any-type falls.**


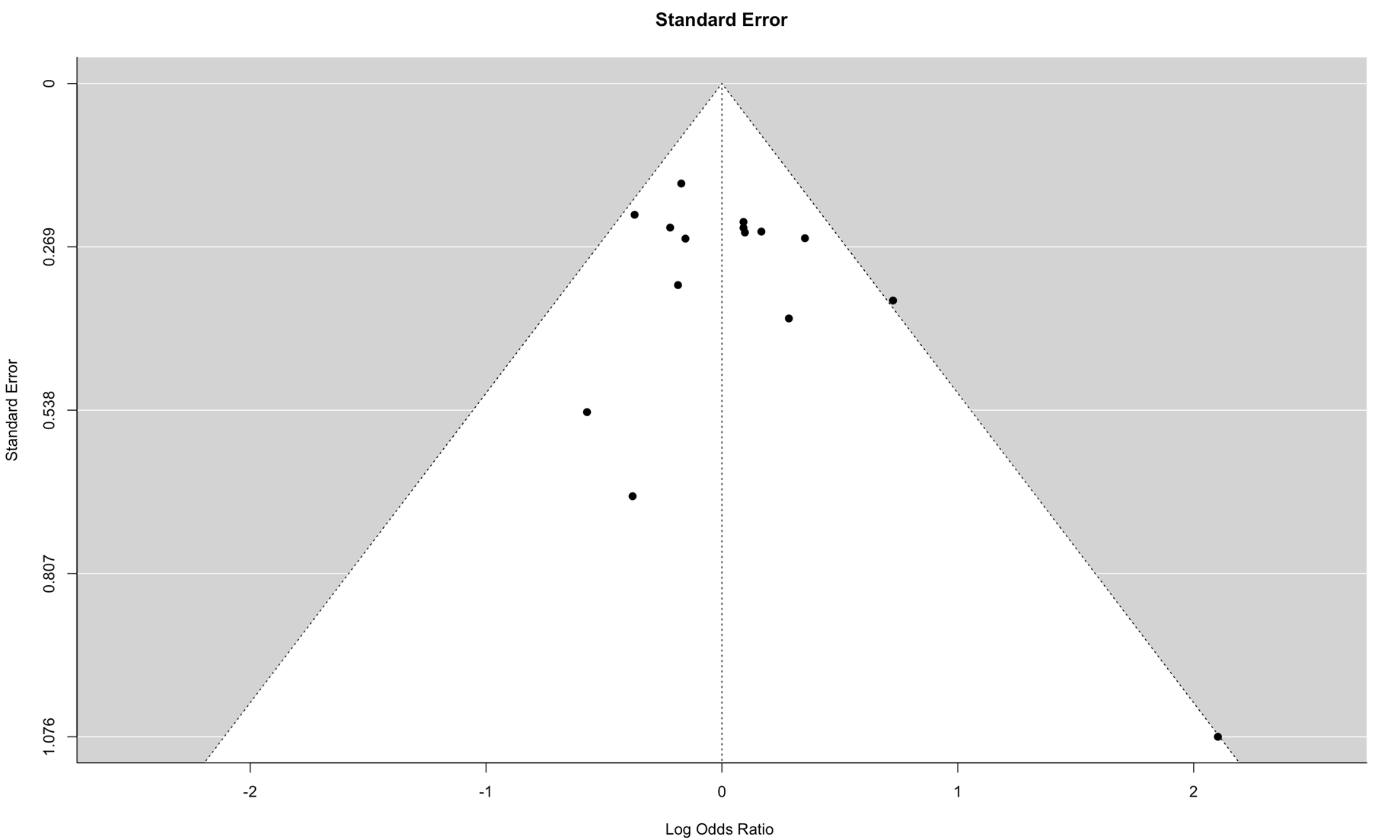


Both visual inspection for asymmetry and the results of Egger’s linear regression test (Z = 1.75, *P* = 0.08) suggest a lack of significant publication bias.

**Appendix F. Sensitivity and subgroup analyses.**

**Figure F1. Forest plot of the association between short 7-item Falls Efficacy Scale International (FES-I) and future any-type falls, excluding the outlier** (Burns et al., 2022)


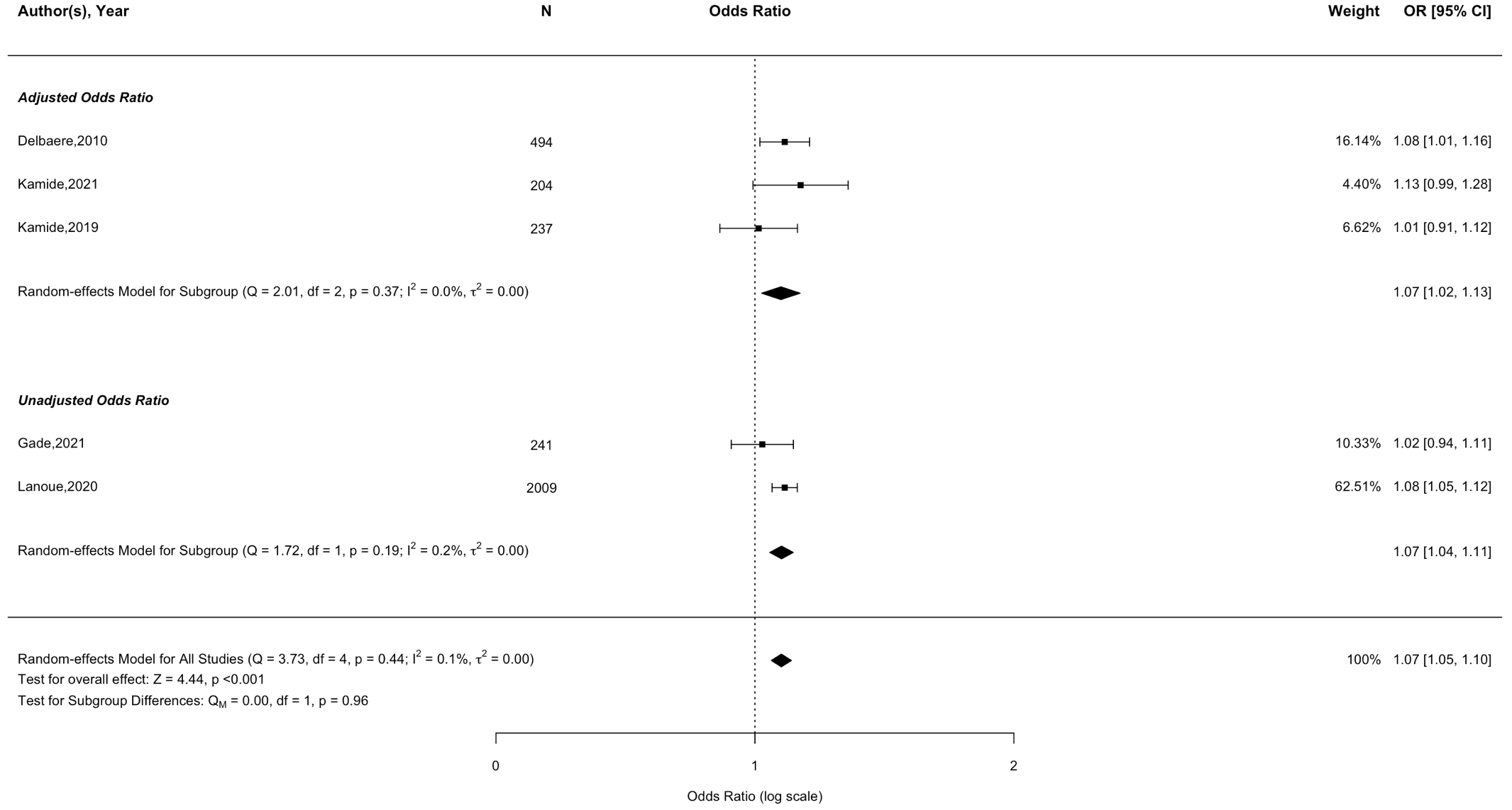


**Figure F2. Forest plot of the association between single-item measures of concerns about falling and future any-type falls, excluding the outlier** (Delbaere et al., 2004)


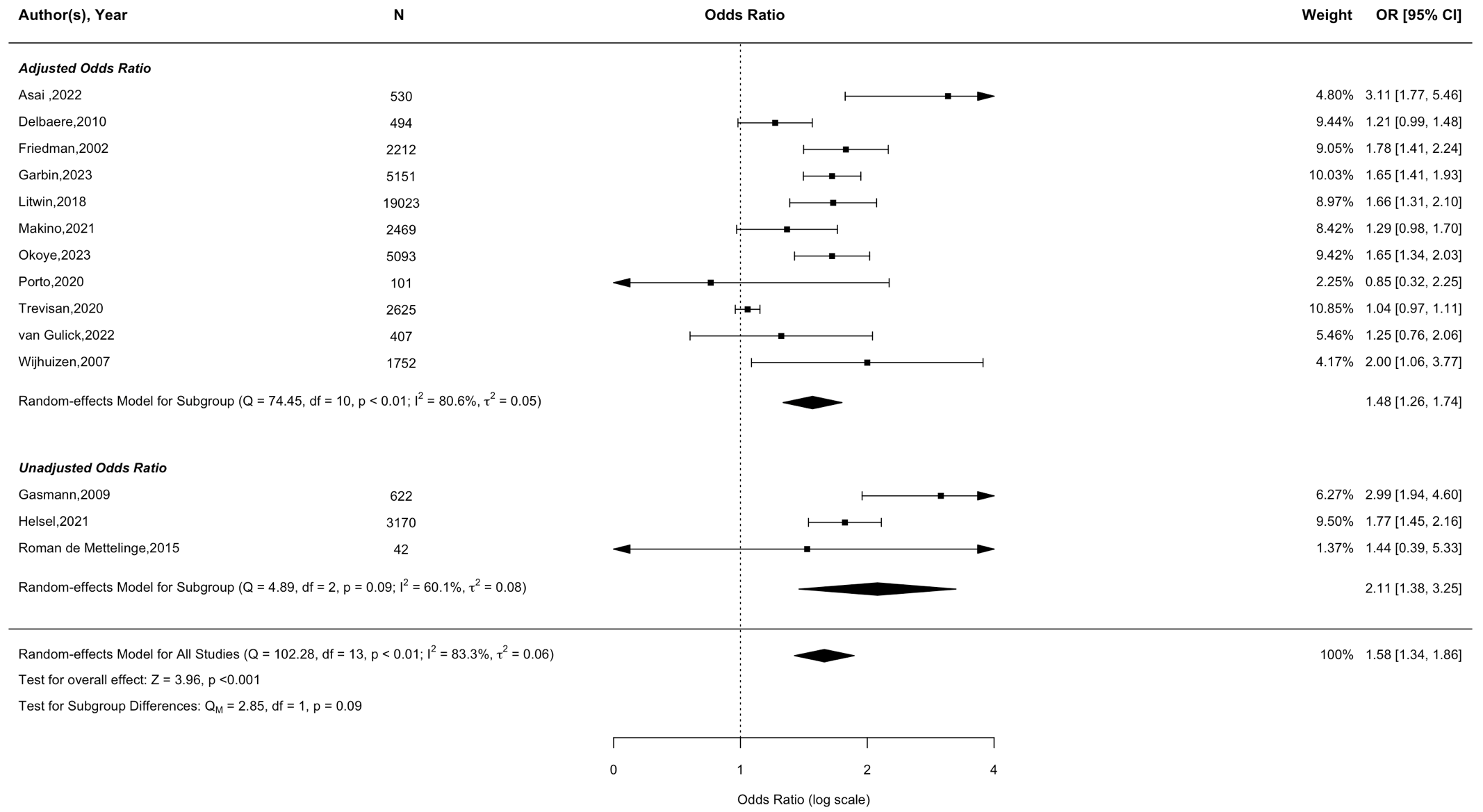


**Figure F3. Forest plot for full 16-item Falls Efficacy Scale International (FES-I) and future any-type falls, separated by risk of bias.**


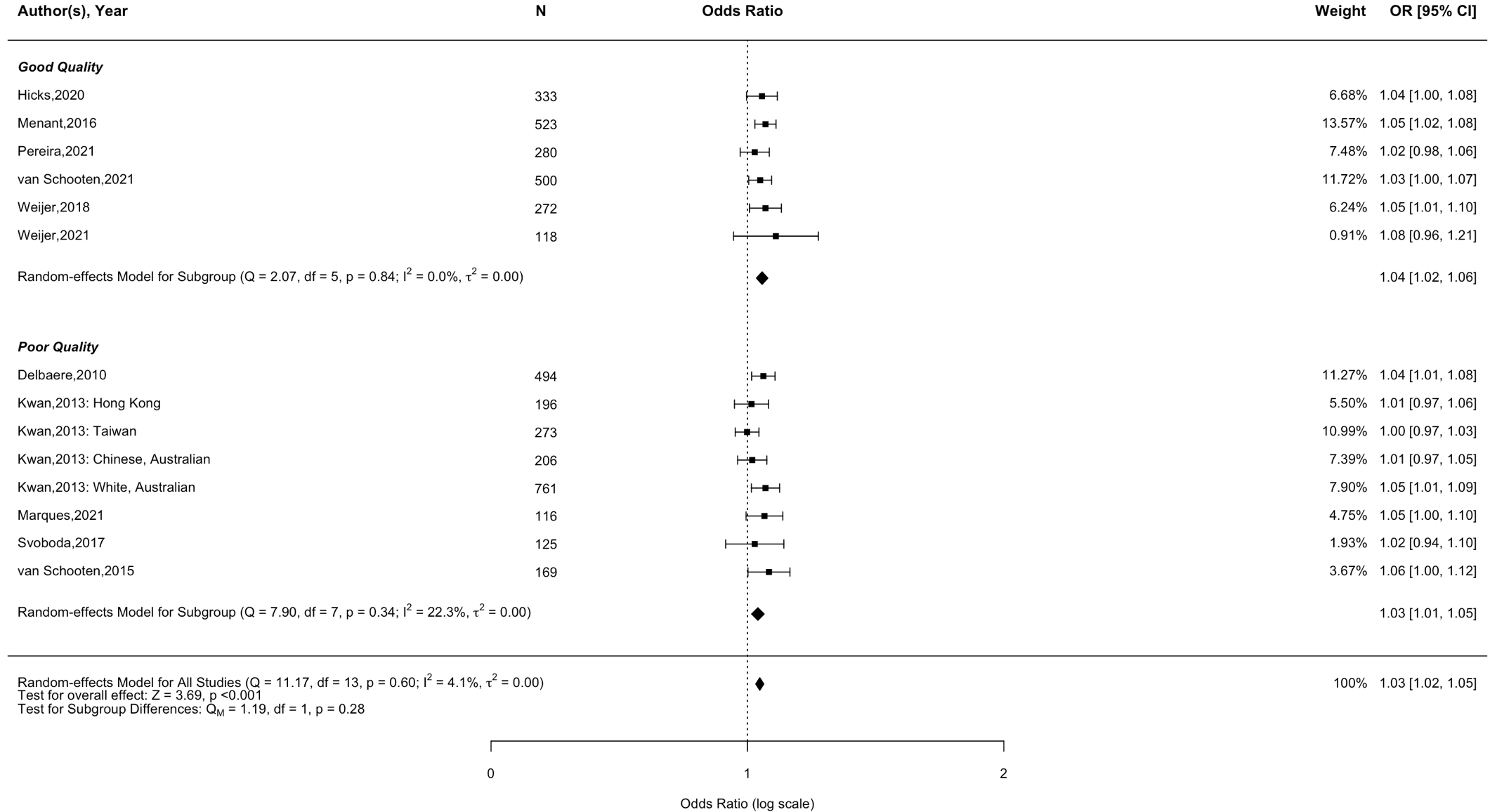


**Figure F4. Forest plot for short 7-item Falls Efficacy Scale International (FES-I) and future any-type falls, separated by risk of bias.**


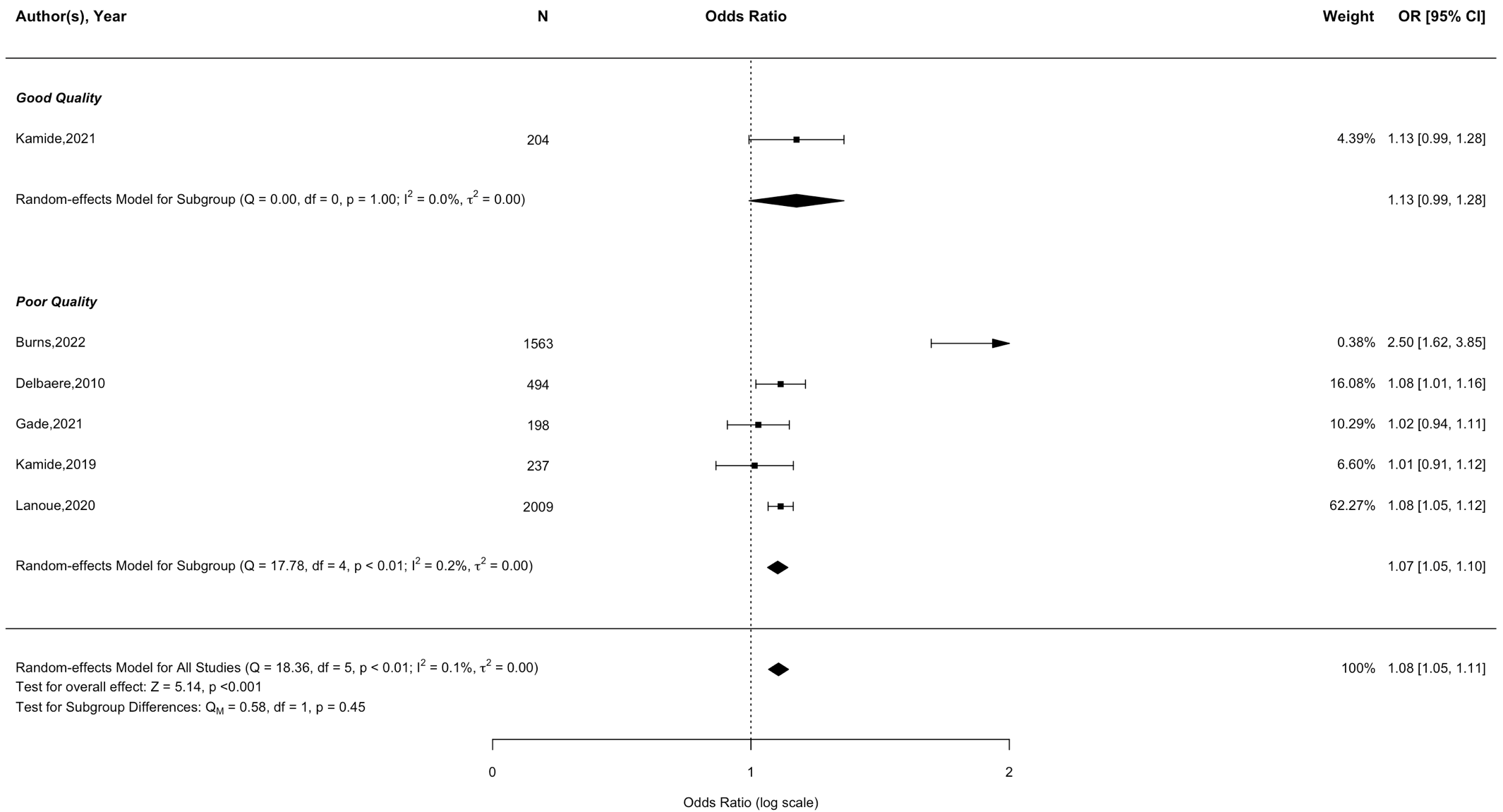


**Figure F5. Forest plot for single-item measures of concerns about falling and future any-type falls, separated by risk of bias.**


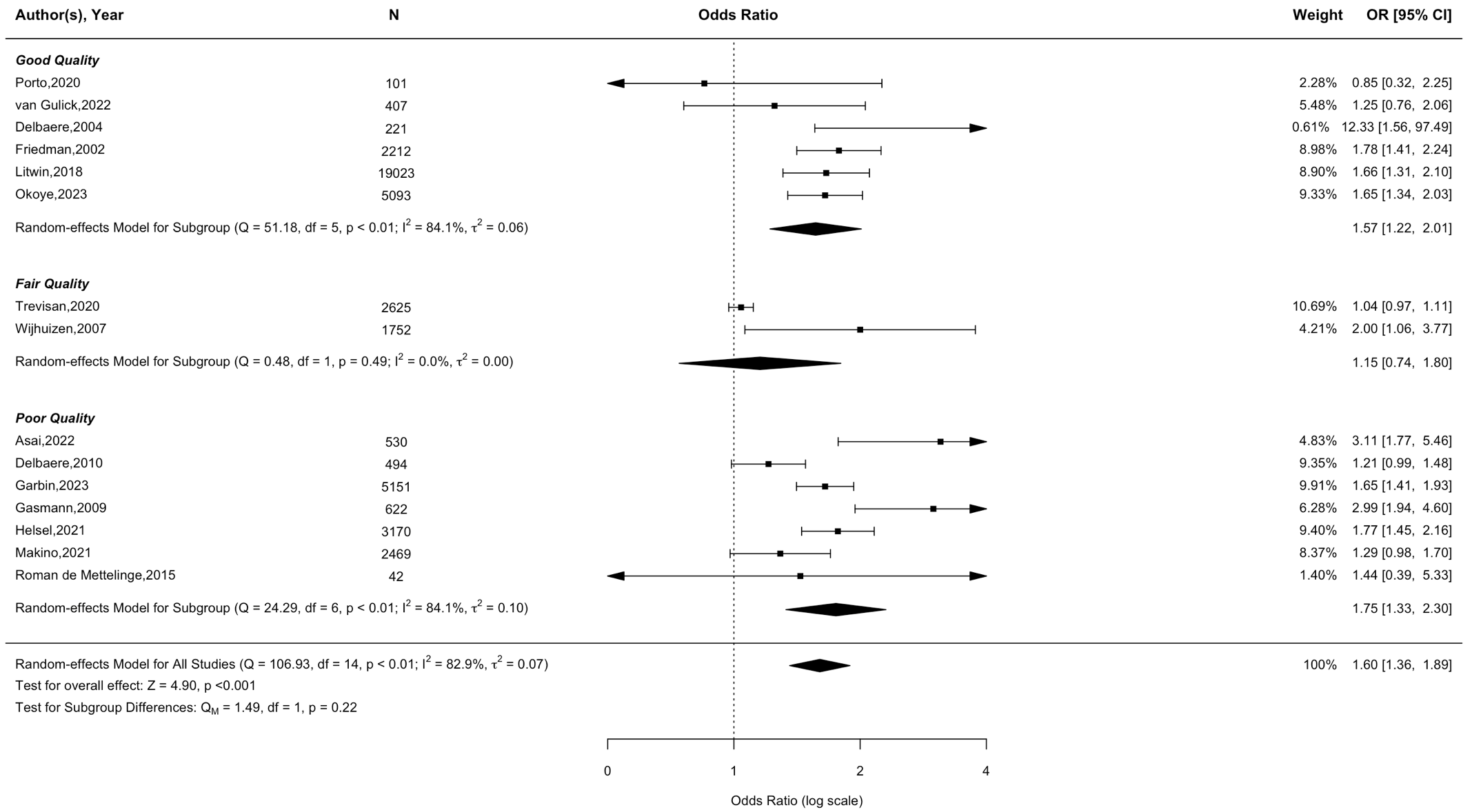


**Figure F6. Forest plot for balance confidence (Activities-Specific Balance Confidence Scale [ABC]) and future any-type falls, separated by risk of bias.**


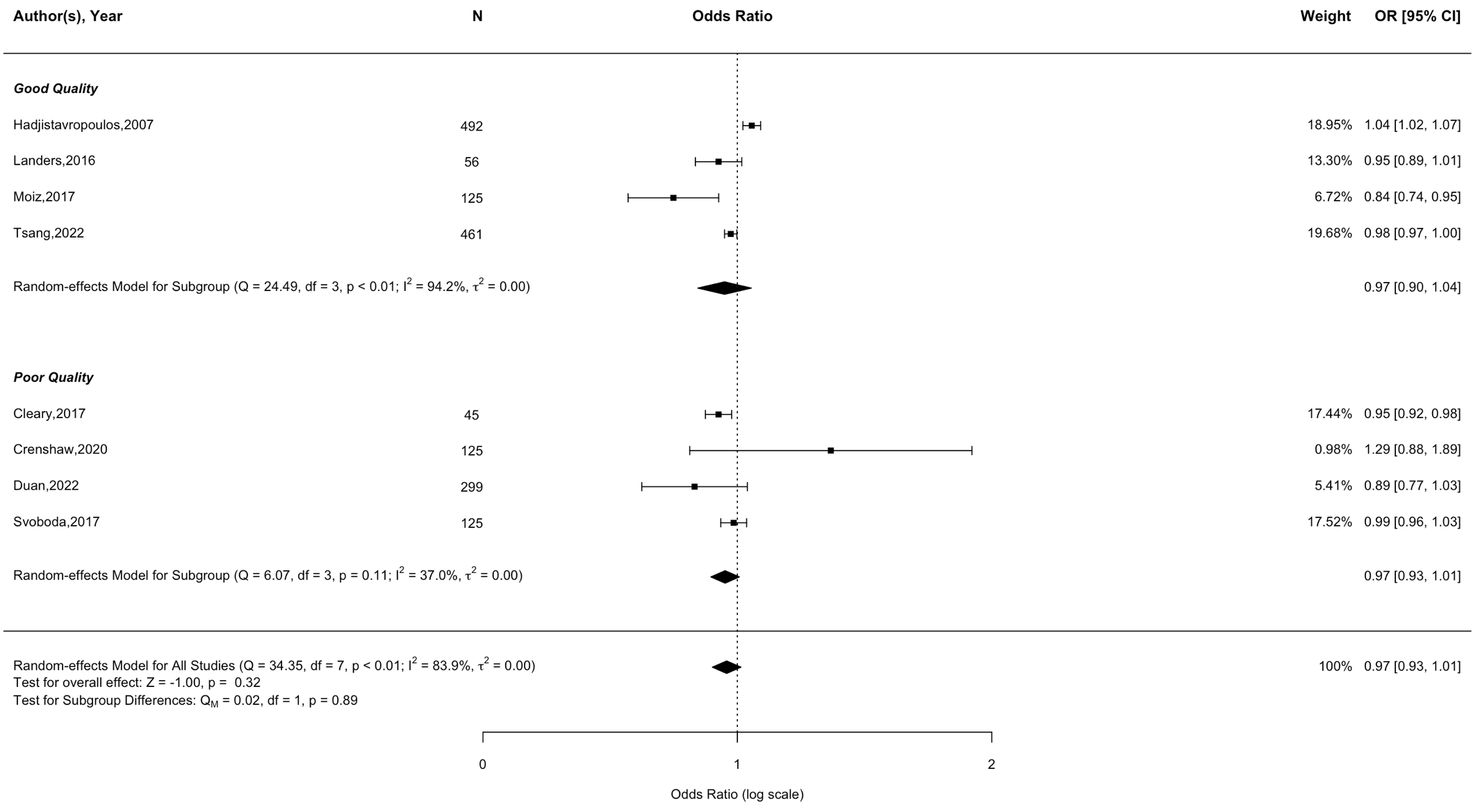


**Appendix G. Risk of bias scoring system.**

**ADAPTED NEWCASTLE - OTTAWA QUALITY ASSESSMENT SCALE COHORT STUDIES**

*Note: A study can be awarded a maximum of one star for each numbered item within the Selection and Outcome categories. A maximum of two stars can be given for comparability.*

**Selection**

**1) Representativeness of the exposed cohort**

a) truly representative of the average older person in the community *

b) somewhat representative of the average older person in the community *

c) selected group of users eg fallers only

d) no description of the derivation of the cohort

**2) Selection of the non-exposed cohort**

a) drawn from the same community as the exposed cohort *

b) drawn from a different source

c) no description of the derivation of the non-exposed cohort

**3) Ascertainment of exposure**

a) Validated multi-item measure *

b) Unvalidated multi-item measure *

c) Single-item measure of concerns about falling

d) No description

**4) Sample size**

a) Justified and satisfactory *

b) Adequately powered to detect a difference (at least 10 participants per variable in final analyses) *

c) Not justified nor adequately powered (see b)

**Comparability**

**1) Comparability of cohorts on the basis of the design or analysis**

a) Study controls for age and sex *

b) Study controls for at least two of: physical function (inc. mobility, balance, gait, etc.) and/or psychological/cognitive function (inc. cognition, anxiety, depression, etc.) and/or previous falls *

c) Study controls for variables, but not those listed in a) or b) d) Does not control for variables

*NOTE: If study contains only males or females, ignore ‘sex’ from option A.*

*NOTE: Possible to score two stars for this domain.*

**Outcome**

**1) Assessment of outcome**

a) Monthly (or more regular) fall diaries and/or telephone calls *

b) Retrospective recall; duration >1 month, and <6 months (e.g. 3 month phone call) *

c) Retrospective recall at the end of follow-up d) No description

**2) Was follow-up long enough for outcomes to occur**

a) yes (6-months or longer) *

b) no (less than 6-months)

**3) Adequacy of follow up of cohorts**

a) complete follow up - all subjects accounted for *

b) subjects lost to follow up unlikely to introduce bias (follow up rate of 80% or greater), or comparison conducted between those lost and not lost *

c) follow up rate < 80% and no description of those lost

d) no statement

**Thresholds for converting the Newcastle-Ottawa scales to AHRQ standards (good, fair, and poor):**

Good quality: 3 or 4 stars in selection domain AND 1 or 2 stars in comparability domain AND 2 or 3 stars in outcome/exposure domain

Fair quality: 2 stars in selection domain AND 1 or 2 stars in comparability domain AND 2 or 3 stars in outcome/exposure domain

Poor quality: 0 or 1 star in selection domain OR 0 stars in comparability domain OR 0 or 1 stars in outcome/exposure domain

**Appendix H. Risk of bias assessment.**

|  |  | **Selection** | | | | **Comparability** | **Outcome** | | |
| --- | --- | --- | --- | --- | --- | --- | --- | --- | --- |
| Study | Overall Rating | Representative of exposed cohort | Selection of non-exposed cohort | Ascertainment of exposure | Sample size |  | Ascertainment of outcome | Follow-up duration | Follow-up adequacy (i.e. missing data) |
| Allali 2017 | **Good** | ***** | ***** | ***** | ***** | ****** | ***** | ***** | ***** |
| Aoyama 2011 | **Poor** |  | ***** | ***** |  |  | ***** | ***** | ***** |
| Asai 2022 | **Poor** | ***** | ***** |  | ***** | ****** |  | ***** |  |
| Burns 2022 | **Poor** | ***** | ***** | ***** | ***** |  | ***** | ***** | ***** |
| Cleary 2017 | **Poor** | ***** | ***** | ***** |  |  | ***** | ***** | ***** |
| Clemson 2015 | **Poor** | ***** | ***** |  | ***** |  |  | ***** | ***** |
| Crenshaw 2020 | **Poor** | ***** | ***** | ***** | ***** |  | ***** | ***** | ***** |
| Cumming 2000 | **Good** | ***** | ***** | ***** | ***** | ****** | ***** | ***** | ***** |
| Delbaere 2004 | **Good** | ***** | ***** | ***** | ***** | ***** | ***** | ***** | ***** |
| Delbaere 2006 | **Poor** | ***** | ***** | ***** | ***** |  | ***** | ***** | ***** |
| Delbaere 2010 | **Poor** | ***** | ***** | ***** | ***** |  | ***** | ***** | ***** |
| de Souza 2019 | **Poor** | ***** | ***** | ***** | ***** | ***** |  | ***** |  |
| Duan 2022 | **Poor** | ***** | ***** | ***** | ***** |  |  | ***** | ***** |
| Faulkner 2009 | **Good** | ***** | ***** |  | ***** | ***** | ***** | ***** | ***** |
| Friedman 2002 | **Good** | ***** | ***** |  | ***** | ***** |  | ***** | ***** |
| Gade 2021 | **Poor** | ***** | ***** | ***** | ***** |  | ***** | ***** | ***** |
| Garbin 2023 | **Poor** | ***** | ***** |  | ***** | ****** |  | ***** |  |
| Gasmann 2009 | **Poor** | ***** | ***** |  | ***** |  |  | ***** |  |
| Hadjistavropoulos 2007 | **Good** | ***** | ***** | ***** | ***** | ***** | ***** | ***** | ***** |
| Helsel 2021 | **Poor** | ***** | ***** |  | ***** |  |  | ***** |  |
| Hicks 2020 | **Good** | ***** | ***** | ***** | ***** | ****** | ***** | ***** | ***** |
| Kamide 2019 | **Poor** | ***** | ***** | ***** | ***** | ****** |  | ***** |  |
| Kamide 2021 | **Good** | ***** | ***** | ***** | ***** | ****** |  | ***** | ***** |
| Kwan 2013 | **Poor** | ***** | ***** | ***** | ***** |  | ***** | ***** | ***** |
| Landers 2016 | **Good** | ***** | ***** | ***** | ***** | ****** |  | ***** | ***** |
| Lanoue 2020 | **Poor** |  | ***** | ***** | ***** |  | ***** | ***** | ***** |
| Lavedan 2018 | **Poor** | ***** | ***** |  | ***** | ****** |  | ***** |  |
| Lim 2021 | **Poor** | ***** | ***** | ***** | ***** |  | ***** | ***** |  |
| Litwin 2018 | **Good** | ***** | ***** |  | ***** | ****** |  | ***** | ***** |
| Lunkinen 1996 | **Good** | ***** | ***** |  | ***** | ***** | ***** | ***** |  |
| Luukinen 1997 | **Poor** |  | ***** |  | ***** |  | ***** | ***** |  |
| Makino 2021 | **Poor** | ***** | ***** |  | ***** | ***** |  | ***** |  |
| Marques 2021 | **Poor** |  | ***** | ***** | ***** |  |  | ***** |  |
| Menant 2016 | **Good** | ***** | ***** | ***** | ***** | ***** | ***** | ***** | ***** |
| Moiz 2017 | **Good** | ***** | ***** | ***** | ***** | ***** | ***** | ***** | ***** |
| Okoye 2023 | **Good** | ***** | ***** |  | ***** | ****** |  | ***** | ***** |
|  |  | **Selection** | | | | **Comparability** | **Outcome** | | |
| Study | Overall Rating | Representative of exposed cohort | Selection of non-exposed cohort | Ascertainment of exposure | Sample size |  | Ascertainment of outcome | Follow-up duration | Follow-up adequacy (i.e. missing data) |
| Pereira 2021 | **Good** | ***** | ***** | ***** | ***** | ****** |  | ***** | ***** |
| Pluijm 2006 | **Good** | ***** | ***** | ***** | ***** | ***** | ***** | ***** | ***** |
| Porto 2020 | **Fair** | ***** | ***** |  |  | ***** | ***** | ***** | ***** |
| Roman de Mettelinge 2015 | **Poor** | ***** | ***** |  | ***** |  | ***** | ***** | ***** |
| Svoboda 2017 | **Poor** | ***** | ***** | ***** | ***** |  | ***** | ***** |  |
| Trevisan 2020 | **Good** | ***** | ***** |  | ***** | ****** |  | ***** | ***** |
| Tromp 2001 | **Poor** | ***** | ***** | ***** | ***** |  | ***** | ***** | ***** |
| Tsang 2022 | **Good** |  | ***** | ***** | ***** | ***** | ***** | ***** | ***** |
| van Gulick 2022 | **Fair** |  | ***** |  | ***** | ***** | ***** | ***** | ***** |
| van Schooten 2015 | **Poor** | ***** | ***** | ***** | ***** |  | ***** | ***** |  |
| van Schooten 2021 | **Good** | ***** | ***** | ***** | ***** | ****** | ***** | ***** | ***** |
| Ward 2015 | **Good** | ***** | ***** | ***** | ***** | ****** | ***** | ***** | ***** |
| Weijer 2018 | **Good** | ***** | ***** | ***** | ***** | ****** | ***** | ***** |  |
| Weijer 2021 | **Good** | ***** | ***** | ***** | ***** | ***** | ***** | ***** | ***** |
| Welmer 2023 | **Good** | ***** | ***** |  | ***** | ****** | ***** | ***** | ***** |
| Wijhuizen 2007 | **Good** | ***** | ***** |  | ***** | ***** | ***** | ***** | ***** |
| Yang 2020 | **Good** |  | ***** | ***** | ***** | ***** | ***** | ***** |  |

| **Appendix I. GRADE certainty of evidence scoring.** | | | | | | | | | | |
| --- | --- | --- | --- | --- | --- | --- | --- | --- | --- | --- |
| **Certainty Assessment** | | | | | | | **No of individuals** | **OR (95% CI)** | **Certainty** | |
| **No of studies** | **Design** | **Risk of bias** | **Inconsistency** | **Indirectness** | **Imprecision** | **Other Considerations** |  |  |  |  |
| **FES-I (16-item)** | | | | | | | | | | |
| 14 (11 articles) | Observational studies | Serious - 8 studies rated as poor quality; 6 studies rated as good quality *(-1)* | No serious inconsistency, I² = 4.1% | No serious indirectness | No serious imprecision | No serious publication bias - No asymmetry evident in funnel plot | 4,366 | 1.03 (1.02, 1.05) | Moderate | ⊕⊕⊕ |
| **Short FES-I (7-item)** | | | | | | | | | | |
| 6 | Observational studies | Serious – 5 studies rated as poor quality; 1 study rated as good quality *(-1)* | No serious inconsistency, I² = 0.1% | No serious indirectness | No serious imprecision | N/A | 4,705 | 1.08 (1.05, 1.11) | Moderate | ⊕⊕⊕ |
| **Single Item CAF** | | | | | | | | | | |
| 15 | Observational studies | Serious – 9 studies rated as poor or fair quality; 6 studies rated as good quality *(-1)* | No serious inconsistency, Large I² (82.9%) but similar point estimates | No serious indirectness | No serious imprecision | No serious publication bias - No asymmetry evident in funnel plot | 43,912 | 1.60 (1.36, 1.89) | Moderate | ⊕⊕⊕ |
| **Balance Confidence (ABC)** | | | | | | | | | | |
| 8 | Observational studies | No serious risk of bias | No serious inconsistency, Large I² (83.9%) but similar point estimates | No serious indirectness | Serious imprecision (95% CI overlaps no effect) *(-1)* | N/A | 1,728 | 0.97 (0.93, 1.01 | Moderate | ⊕⊕⊕ |

GRADE scoring is in italics.

**Appendix J. PRISMA guidelines checklist**

| **Section and Topic** | **Item #** | **Checklist item** | **Location where item is reported** |
| --- | --- | --- | --- |
| **TITLE** | | |  |
| Title | 1 | Identify the report as a systematic review. | Page 1 |
| **ABSTRACT** | | |  |
| Abstract | 2 | See the PRISMA 2020 for Abstracts checklist. | Page 2 |
| **INTRODUCTION** | | |  |
| Rationale | 3 | Describe the rationale for the review in the context of existing knowledge. | Intro, paragraph 1 |
| Objectives | 4 | Provide an explicit statement of the objective(s) or question(s) the review addresses. | Intro, final paragraph |
| **METHODS** | | |  |
| Eligibility criteria | 5 | Specify the inclusion and exclusion criteria for the review and how studies were grouped for the syntheses. | Methods, ‘Eligibility Criteria and Study Selection’ section |
| Information sources | 6 | Specify all databases, registers, websites, organisations, reference lists and other sources searched or consulted to identify studies. Specify the date when each source was last searched or consulted. | Methods, ‘Search Strategy and Information Sources’ section |
| Search strategy | 7 | Present the full search strategies for all databases, registers and websites, including any filters and limits used. | Supplementary Appendix A |
| Selection process | 8 | Specify the methods used to decide whether a study met the inclusion criteria of the review, including how many reviewers screened each record and each report retrieved, whether they worked independently, and if applicable, details of automation tools used in the process. | Methods, ‘Eligibility Criteria and Study Selection’ section |
| Data collection process | 9 | Specify the methods used to collect data from reports, including how many reviewers collected data from each report, whether they worked independently, any processes for obtaining or confirming data from study investigators, and if applicable, details of automation tools used in the process. | Methods, ‘Data Extraction’ section |
| Data items | 10a | List and define all outcomes for which data were sought. Specify whether all results that were compatible with each outcome domain in each study were sought (e.g. for all measures, time points, analyses), and if not, the methods used to decide which results to collect. | Methods, ‘Data Extraction’ section |
|  | 10b | List and define all other variables for which data were sought (e.g. participant and intervention characteristics, funding sources). Describe any assumptions made about any missing or unclear information. | Methods, ‘Data Extraction’ section |
| Study risk of bias assessment | 11 | Specify the methods used to assess risk of bias in the included studies, including details of the tool(s) used, how many reviewers assessed each study and whether they worked independently, and if applicable, details of automation tools used in the process. | Methods, ‘Risk of Bias and Quality Assessment’ section |
| Effect measures | 12 | Specify for each outcome the effect measure(s) (e.g. risk ratio, mean difference) used in the synthesis or presentation of results. | Methods, ‘Statistical Analysis’ section |
| Synthesis methods | 13a | Describe the processes used to decide which studies were eligible for each synthesis (e.g. tabulating the study intervention characteristics and comparing against the planned groups for each synthesis (item #5)). | Methods, ‘Data Extraction’ and ‘Statistical Analysis’ section |
|  | 13b | Describe any methods required to prepare the data for presentation or synthesis, such as handling of missing summary statistics, or data conversions. | Methods, ‘Statistical Analysis’ section |
|  | 13c | Describe any methods used to tabulate or visually display results of individual studies and syntheses. | Methods, ‘Statistical Analysis’ section |
|  | 13d | Describe any methods used to synthesize results and provide a rationale for the choice(s). If meta-analysis was performed, describe the model(s), method(s) to identify the presence and extent of statistical heterogeneity, and software package(s) used. | Methods, ‘Statistical Analysis’ section |
|  | 13e | Describe any methods used to explore possible causes of heterogeneity among study results (e.g. subgroup analysis, meta-regression). | Methods, ‘Statistical Analysis’ section |
|  | 13f | Describe any sensitivity analyses conducted to assess robustness of the synthesized results. | Methods, ‘Statistical Analysis’ section |
| Reporting bias assessment | 14 | Describe any methods used to assess risk of bias due to missing results in a synthesis (arising from reporting biases). | Methods, ‘Risk of Bias and Quality Assessment’ section |
| Certainty assessment | 15 | Describe any methods used to assess certainty (or confidence) in the body of evidence for an outcome. | Methods, ‘Grading of Recommendations, Assessment, Development and Evaluation’ section |
| **RESULTS** | | |  |
| Study selection | 16a | Describe the results of the search and selection process, from the number of records identified in the search to the number of studies included in the review, ideally using a flow diagram. | Results, paragraph 1 |
|  | 16b | Cite studies that might appear to meet the inclusion criteria, but which were excluded, and explain why they were excluded. | Results, paragraph 1 |
| Study characteristics | 17 | Cite each included study and present its characteristics. | Supplementary Appendix B, C & D |
| Risk of bias in studies | 18 | Present assessments of risk of bias for each included study. | Supplementary Appendix H |
| Results of individual studies | 19 | For all outcomes, present, for each study: (a) summary statistics for each group (where appropriate) and (b) an effect estimate and its precision (e.g. confidence/credible interval), ideally using structured tables or plots. | Supplementary Appendix B |
| Results of syntheses | 20a | For each synthesis, briefly summarise the characteristics and risk of bias among contributing studies. | Results, each paragraph where data is synthesised |
|  | 20b | Present results of all statistical syntheses conducted. If meta-analysis was done, present for each the summary estimate and its precision (e.g. confidence/credible interval) and measures of statistical heterogeneity. If comparing groups, describe the direction of the effect. | Figures 2-5; Supplementary Appendix F |
|  | 20c | Present results of all investigations of possible causes of heterogeneity among study results. | Supplementary Appendix F |
|  | 20d | Present results of all sensitivity analyses conducted to assess the robustness of the synthesized results. | Supplementary Appendix F |
| Reporting biases | 21 | Present assessments of risk of bias due to missing results (arising from reporting biases) for each synthesis assessed. | Results, ‘FES-I’ and ‘single-item assessment’ sections; Supplementary Appendix E |
| Certainty of evidence | 22 | Present assessments of certainty (or confidence) in the body of evidence for each outcome assessed. | Supplementary Appendix I |
| **DISCUSSION** | | |  |
| Discussion | 23a | Provide a general interpretation of the results in the context of other evidence. | Discussion, first four paragraphs |
|  | 23b | Discuss any limitations of the evidence included in the review. | Discussion, ‘Methodological limitations of studies included in the review’ section |
|  | 23c | Discuss any limitations of the review processes used. | Discussion, ‘Strengths and limitations of this review’ section |
|  | 23d | Discuss implications of the results for practice, policy, and future research. | Discussion, ‘Clinical recommendations’ section |
| **OTHER INFORMATION** | | |  |
| Registration and protocol | 24a | Provide registration information for the review, including register name and registration number, or state that the review was not registered. | Methods, ‘Registration and Protocol’ section |
|  | 24b | Indicate where the review protocol can be accessed, or state that a protocol was not prepared. | Methods, ‘Registration and Protocol’ section |
|  | 24c | Describe and explain any amendments to information provided at registration or in the protocol. | Methods, ‘Risk of Bias and Quality Assessment’ section |
| Support | 25 | Describe sources of financial or non-financial support for the review, and the role of the funders or sponsors in the review. | ‘Support’ section |
| Competing interests | 26 | Declare any competing interests of review authors. | ‘Competing Interests’ section |
| Availability of data, code and other materials | 27 | Report which of the following are publicly available and where they can be found: template data collection forms; data extracted from included studies; data used for all analyses; analytic code; any other materials used in the review. | ‘Availability of data, code, and other materials’ section |

*From:*  Page MJ, McKenzie JE, Bossuyt PM, Boutron I, Hoffmann TC, Mulrow CD, et al. The PRISMA 2020 statement: an updated guideline for reporting systematic reviews. BMJ 2021;372:n71. doi: 10.1136/bmj.n7
